# Alizarin Dye: Toxicity, Genotoxicity, and Histopathological Alterations in Model Organisms

**DOI:** 10.1002/em.70046

**Published:** 2026-04-24

**Authors:** Amanda Rocha Rodrigues, Gabriela Cristina Fonseca Almeida, Natália Oliveira de Farias, Anjaina Fernandes de Albuquerque, Adria Caloto de Oliveira, Jessica Camila Miranda Cardoso, Gabriely Fernanda Groto Militão, Inês Moutinho Cabral, Catarina A. Faustino, João D. Vitorino, Marina Tenório Botelho, Pedro M. Costa, Gisela de Aragão Umbuzeiro

**Affiliations:** ^1^ Faculdade de Tecnologia Universidade Estadual de Campinas Limeira Brazil; ^2^ Associate Laboratory i4HB Institute for Health and Bioeconomy NOVA School of Science and Technology, NOVA University of Lisbon Caparica Portugal; ^3^ UCIBIO Applied Molecular Biosciences Unit, Department of Life Sciences NOVA School of Science and Technology, NOVA University of Lisbon Caparica Portugal

**Keywords:** anthraquinones, DNA damage, ecotoxicity, gonads, hemocytes, sperm cells

## Abstract

Alizarin is an anthraquinone red dye from natural or synthetic sources, widely used in textiles. Effluents of this activity can contain residual dyes, which may contaminate the aquatic environment. Studies report alizarin's aquatic toxicity, mutagenic, and carcinogenic effects. This study aimed to complement the aquatic toxicity evaluation and confirm its ability to cause genotoxicity in alternative models. Acute toxicity was performed with crustaceans, mussels, and fish embryos, while chronic toxicity was assessed in algae. Light effects on toxicity were evaluated using *Daphnia similis* . Histopathological effects on the gonads of *Mytilus galloprovincialis* and somatic mutations and sperm genotoxicity in *Parhyale hawaiensis* were investigated. Mutagenicity was confirmed using a miniaturized Ames test. The effect concentration 50% (EC_50_) for *D. similis* was 90.3, 105, and 68.6 μg L^−1^ for photoperiod (16 h light:8 h dark), light and dark, respectively. For *Danio rerio* embryos, the lethal concentration 50% (LC_50_) was 45.8 μg L^−1^, and an EC_10_ of 20.8 μg L^−1^ was calculated for sublethal effects. In vivo exposures caused alterations in the digestive gland and gonads of *M. galloprovincialis* , even in a short‐term exposure, and increased the frequency of micronuclei and DNA damage in hemocytes and spermatozoids, respectively, of *P. hawaiensis* . It was mutagenic in the miniaturized Ames test using strain TA1537 (10% and 30% S9). Alizarin can be classified as a Category 1 acute aquatic toxicity according to the globally harmonized system (GHS). Due to adverse histopathological and DNA effects on reproductive systems in model organisms, it is considered a potential germ cell mutagen.

## 1 Introduction

There is a tendency to replace synthetic dyes with colorants of natural origin (Blois 2025). In the fashion industry, alizarin obtained from plants is considered an important red dye. Regardless of its natural origin, there are concerns related to its potential health and environmental impacts.

Alizarin can be obtained from natural or synthetic sources (Do et al. 2023). When derived from natural sources, it is extracted from the roots of the *Rubia tinctorum* plant and is known as madder, a rich source of various anthraquinones, with alizarin as the main component (Bechtold and Mussak 2009). In the plant, alizarin is predominantly found in its glycoside form (Ford et al. 2015; Khan et al. 2023). Alizarin, derived from madder, is considered today a strategic product for sustainable textile dyeing (Ozdemir et al. 2025) and is mainly used in its aglycone form. The aglycone alizarin structure makes it more suitable for dyeing applications (Derksen et al. 2003), including in waterless dyeing techniques using supercritical CO_2_ (Schmidt et al. 2025). Alizarin, in its aglycone form, shares the same chemical structure, regardless of synthetic or natural origin.

Alizarin in its aglycone form has been shown to be toxic to plants, algae, and *Danio rerio* embryos (Babu et al. 2005; Marwood et al. 2003; Knecht et al. 2013). As all anthraquinones, alizarin is expected to have photoinduced toxicity; however, when the microcrustacean *Daphnia magna* was exposed to alizarin (24 h) both in the dark and under simulated solar light, no toxic effects were observed (Wang et al. 2009). On the other hand, a madder extract (containing 89.9% alizarin) was revealed to be toxic to algae and microcrustacean (Freeman et al. 2021) (detailed information see Supporting Information Table S1).

The mutagenicity of alizarin in its aglycone form has been already reported in the literature. Alizarin was mutagenic in the *Salmonella*/microsome assay, and it requires metabolic activation (S9) to exert its effect (Tikkanen et al. 1983; Westendorf et al. 1990; Liberman et al. 1982) (detailed information see Supporting Information I—Table S2a). It was recognized that alizarin is an aryl hydrocarbon receptor (AhR) agonist (Lu et al. 2022). Alizarin increased CYP1A1 mRNA expression in HepG2 and Hepa‐1c1c7 cells via AhR activation, evidenced by AhR nuclear translocation (Lu et al. 2022). In experiments in vivo with vertebrates, aglycone alizarin induced renal preneoplastic lesions in 6‐week‐old male F344 rats after exposure of 23 weeks (Westendorf et al. 1990; Blömeke et al. 1992; Inoue et al. 2009) (detailed info see Supporting Information I—Table S2b). Anthraquinone showed carcinogenicity in the kidneys and urinary bladder of F344 rats exposed for 2 years (National Toxicology Program 2005). Emodin, an anthraquinone similar to alizarin, showed no evidence of carcinogenicity in F344 rats exposed for 2 years (National Toxicology Program 2001).

Although no occurrence data of alizarin was found in the environment, it is expected to increase due to the great interest of this compound as a natural dye. Therefore, there is a need for a broader investigation into the adverse effects of alizarin in different test systems, exploring multiple endpoints. The aim of this study was:

1. complement the existing toxicity data using aquatic model species from different trophic levels and verify its possible photoinduced effects;
2. assess its genotoxicity in in vivo experiments, evaluating somatic cells (hemocytes) and sperm cells (spermatozoids) in the model organism, *Parhyale hawaiensis* ;
3. confirm its mutagenicity in vitro using a Salmonella/microsome miniaturized protocol (Microplate Agar); and
4. verify histopathological alterations in different organs in the *Mytilus* model.

## 2 Materials and Methods

### 2.1 Dye

Alizarin dye in its aglycone form (CAS number: 72‐48‐0, molecular weight 240.21 g mol^−1^, purity 97.1%, obtained from Tokyo Chemical Industry) was dissolved in dimethyl sulfoxide (DMSO) because of its poor solubility in water (Tran et al. 2024). In this work, we empirically calculated its solubility in DMSO as 1.93 g L^−1^. To select the maximum concentration to be tested, we followed the OECD recommendations (OECD 2019) and used a maximum of 0.01% DMSO in the test media for toxicity tests with *R. subcapitata*, *D. similis* , *D. rerio* , and *P. hawaiensis* . Therefore, the maximum concentration tested was 193 μg L^−1^. Maximum DMSO concentration was increased to 0.1% for the acute test and the assays directed to histopathological analysis with *M. galloprovincialis* , comet assay, and micronucleus test based on the absence of effects observed in previous experiments.

### 2.2 Rationale of the Organisms and Endpoints Used in This Work

All organisms used in this study are established models in environmental research. Aquatic toxicity was assessed using algae, microcrustaceans, and fish freshwater species (*Raphidocelis subcapitata*, *Daphnia similis* , and *Danio rerio* embryos) and two marine species, the amphipod *Parhyale hawaiensis* and the bivalve *Mytilus galloprovincialis* . The specific methodologies used for the tests are described in Supporting Information II. Mutagenic effects were evaluated in vitro using the miniaturized Salmonella/microsome assay (Microplate Agar) and in vivo using the micronucleus test in hemocytes and the comet assay in sperm cells of *P. hawaiensis* . Histological analyses were conducted on the gonads and digestive glands of *Mytilus galloprovincialis*. Experimental rationale and endpoints used in this study are summarized in Table 1.

**TABLE 1:** Information on the model organisms and endpoints used in this work.

Model organisms	Description	Endpoints
Freshwater algae | *Raphidocelis subcapitata* 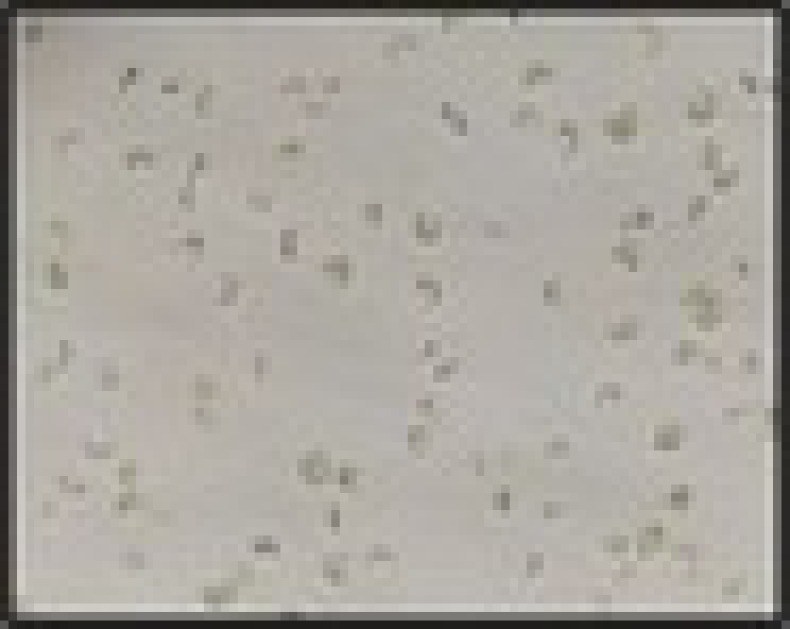	Algae are primary producers essential to aquatic life. *Raphidocelis subcapitata* is used as a standard test organism, following the OECD 201 protocol (OECD 2011)	Growth inhibition
Freshwater crustacean | *Daphnia similis* 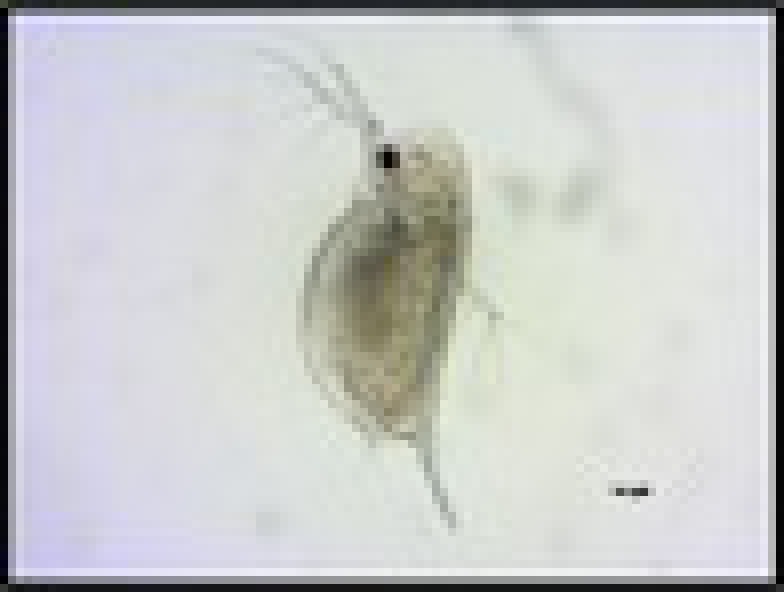	Daphnids are key species in aquatic food webs, characterized by their short generational time and parthenogenetic life cycle. They serve as important indicators of water quality due to their high sensitivity to environmental changes (Silva et al. 2025). Standardized acute toxicity tests have been established for this organism (ABNT 2022; OECD 2004)	Immobilization
Freshwater embryos fish | *Danio rerio* 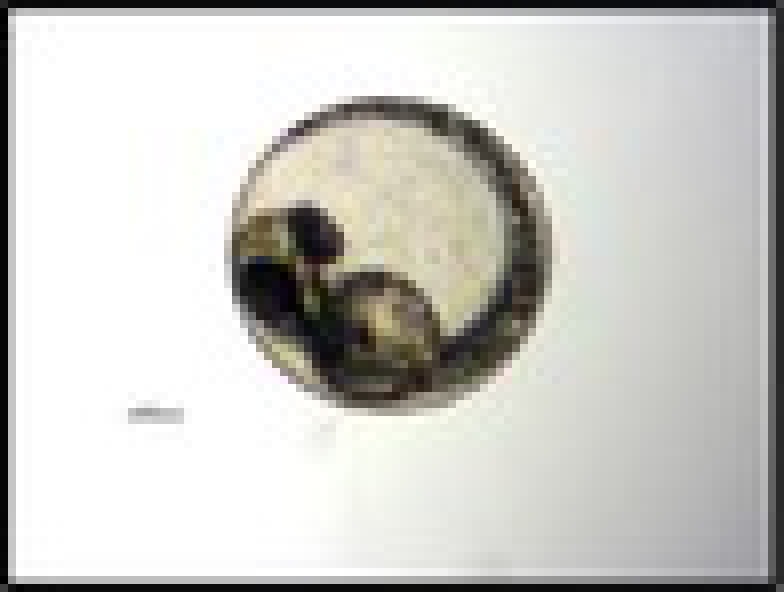	*Danio rerio* fish is a secondary consumer widely cultivated in ecotoxicology laboratories due to its rapid embryonic development, enabling the analysis of toxic effects on the embryo, as well as being the most sensitive stage to adverse effects (Huang et al. 2021). A standardized acute toxicity test for *D. rerio* embryos has been established (OECD 2013)	Lethality
Marine crustacean | *Parhyale hawaiensis* 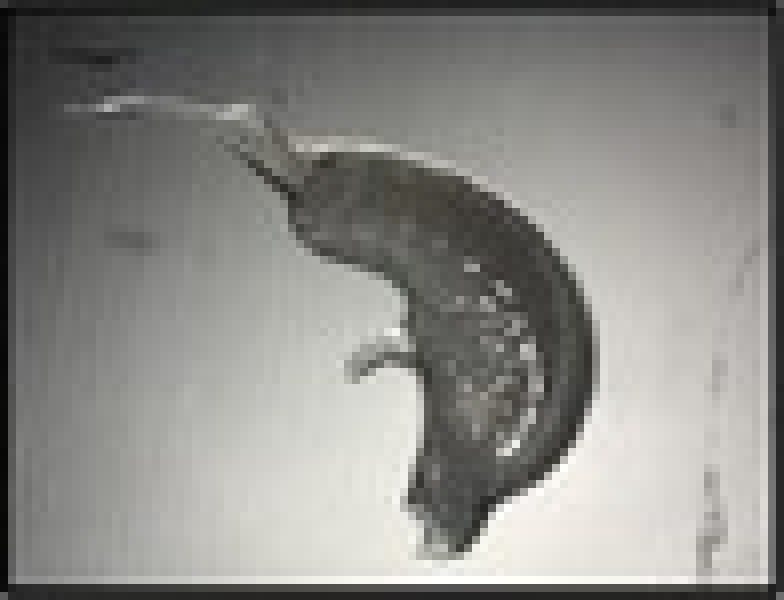	*Parhyale hawaiensis* is an epibenthic, detritivorous organism with a circumtropical distribution that plays a key role in the food chain and is considered a good organism for ecotoxicological testing (dos Santos et al. 2022). Protocols have been established for acute and chronic testing, as well as genotoxicity and mutagenicity testing with this organism (Artal et al. 2017; Vacchi et al. 2019; Botelho and Umbuzeiro 2024; Botelho et al. 2022)	Lethality Chromosome mutations in hemocytes (micronucleus test) DNA damage in testis (comet assay)
Marine mussel | *Mytilus galloprovincialis* 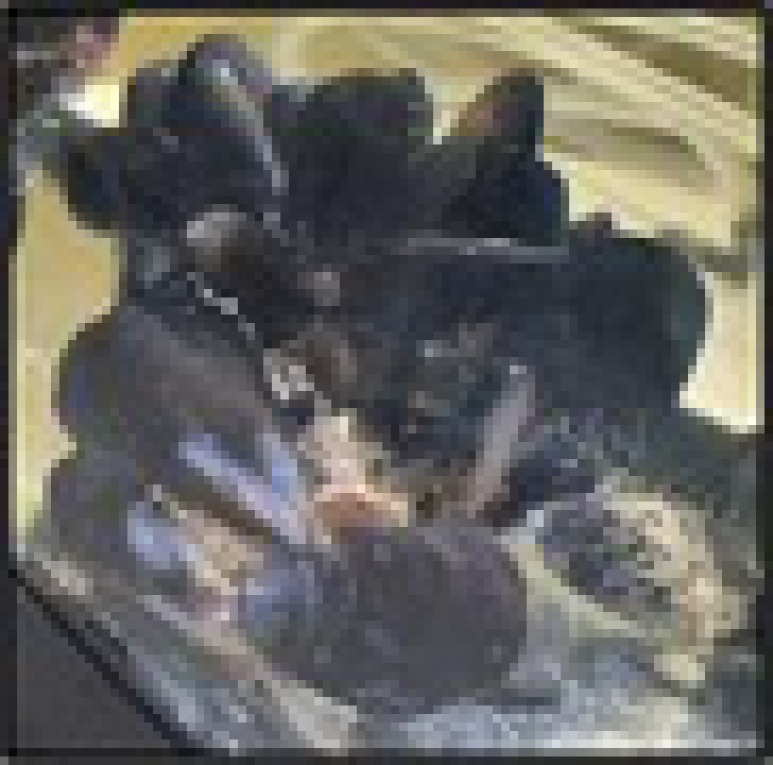	Bivalve mussels are sessile filter‐feeding organisms that reflect the environmental conditions of their habitat as they can accumulate pollutants present in the water (Beyer et al. 2017). They are ecologically and economically important, possess a relatively simple anatomical structure, and are easy to maintain under laboratory conditions. Therefore, species such as *Mytilus galloprovincialis* represent valuable models for both in situ biomonitoring and toxicity testing assays (Beyer et al. 2017; Larguinho et al. 2014)	Lethality Histopathological analysis

### 2.3 Toxicity Tests

Detailed protocols and the results' interpretation for each test can be found in the Supporting Information II (Toxicity Tests Methodology).

Several organisms were used for the standardized toxicity tests: *Raphidocelis subcapitata* (chronic, 72 h; OECD 2011), *Daphnia similis* (acute, 48 h, different light conditions; OECD 2004), *Danio rerio* (embryotoxicity, 168 h; OECD 2013), *Parhyale hawaiensis* (acute, 96 h; Artal et al. 2017), and *Mytilus galloprovincialis* (acute, 72 h). In all tests, the concentrations causing effects in 50% of the population were calculated, specifically: the inhibition concentration (IC_50_), effect concentration (EC_50_), and lethal concentration (LC_50_).

*Raphidocelis subcapitata*, *D. similis* , *D. rerio* , and *P. hawaiensis* were cultivated in the Laboratory of Ecotoxicology and Genotoxicity (LAEG) at Universidade Estadual de Campinas (UNICAMP), Limeira, Brazil. The sensitivity of the cultures was routinely evaluated with NaCl for *R. subcapitata* and *D. similis* and ZnSO_4_ for *P. hawaiensis* (control charts can be found in Supporting Information I—Figures S1–S3). Only cultures that responded within the acceptable sensitivity range were used in the tests. For fish embryo tests, 3,4‐dichloroaniline (3,4‐DCA) was included as a positive control in each experiment and used to validate the results.

*Mytilus galloprovincialis* was collected at Costa de Caparica, W. Portugal, and acclimated in the SeaTox Lab at NOVA School of Science and Technology of the NOVA University of Lisbon, Portugal.

The reliability and relevance of the data generated in all toxicity tests were evaluated using the Criteria for Reporting and Evaluating Ecotoxicity Data (CRED) to ensure the quality of the ecotoxicity data used for regulatory and decision‐making processes (Moermond et al. 2016). To evaluate the data, 20 reliability and 13 relevance criteria were applied for all toxicity tests performed in this study.

### 2.4 Histopathological Analyses

#### 2.4.1 *Mytilus galloprovincialis*

Six mussels per condition were exposed to alizarin at concentrations of 241.25, 482.5, 965, and 1930 μg L^−1^ for 72 h, with daily renewal of the exposure solution. Histopathological analysis was performed according to Costa (2018). After 72 h of exposure, the organisms were fixed in two steps. (1) By immersion and intravalvar injection of Bouin's fixative (formalin, acetic acid and picric acid), under vacuum for 1 h. (2) The whole soft body was removed and immersed in fresh Bouin's solution for a further 24 h. The samples were afterwards washed in water and dehydrated in a progressive series of ethanol (70%–100%). Intermediate infiltration was done with xylenes, preceding embedding in molten paraffin. Sample processing was performed in a Shandon Pathcentre tissue processor (Thermo Fisher Scientific). Five‐μm sections were produced using a RM2245 microtome (Leica Biosystems). The slides were stained with hematoxylin and eosin and mounted with DPX resin. Histopathological analysis was conducted using a DM 2500 model microscope (Leica Microsystems) and a MC 190 HD camera (Leica Microsystems). The identification of traits was made according to Cuevas et al. (2015) and Costa (2018).

### 2.5 Genotoxic Effects

#### 2.5.1 Micronucleus Test With *Parhyale hawaiensis* Hemocytes

To determine the concentrations used in the micronucleus test, a preliminary test was performed with *P. hawaiensis* neonates (≤ 7 days old). The organisms were exposed for 96 h to alizarin (193, 643, 1930, 6430, and 19,300 μg L^−1^) under controlled conditions (temperature 24°C ± 2°C and photoperiod 16 h light:8 h dark) in a 96‐well microplate, using one neonate per well. The mortality was assessed under a stereomicroscope (Stemi, 2000‐C, Carl Zeiss, Oberkochen, Germany). The concentrations used in the micronucleus test were defined by 482.5, 965, and 1930 μg L^−1^ of alizarin.

The micronucleus test was conducted with *P. hawaiensis* hemocytes, following Botelho et al. (2022). Twelve adult organisms (8 months) per condition were exposed to alizarin for 96 h. Zinc sulfate (1.5 mg L^−1^ as Zinc) was used as the positive control, and DMSO 0.1% as the negative control. The hemolymph was collected with a glass capillary inserted into the 2nd or 3rd segment of the adult organism and transferred to the vial with 20 μL artificial seawater. This suspension was transferred to a slide and placed in a humid chamber for 15 min. Then, the slides were washed with artificial seawater, covered with formaldehyde 1:10, and placed in a humid chamber for 15 min. Once dry, the slides were immersed in methanol for 10 min and then stained with 10% Giemsa for 20 min. A total of 500 cells were observed per slide under an optical microscope at ×1000 and the number of micronuclei was counted.

#### 2.5.2 DNA Strand Breakage Assessment on *Parhyale hawaiensis* Sperm Cells

The alkaline Comet assay was conducted on *P. hawaiensis* sperm following Botelho and Umbuzeiro (2024). Sixteen adult organisms (8 months) per condition were exposed to alizarin concentrations (77.2, 386, and 1930 μg L^−1^) for 96 h. Ethyl methanesulfonate (EMS) 2 mM and DMSO 0.1% were used as positive and negative controls, respectively.

The organisms were anesthetized with 0.06% clove oil solution in reconstituted seawater prior to dissection. The dissection was performed under a stereomicroscope using tweezers to remove the testes. The testes were placed on a concave slide with 10 μL of reconstituted seawater and torn apart to release sperm cells. The sperm cell suspension was prepared using a pool of testes from two males in a vial containing 10 μL of cold reconstituted seawater. This vial was vortexed for 10 s. Fifty microliters of 1.0% low melting point agarose prepared in Kenny's salt solution (0.4 M NaCl, 9 mM KCl, 0.7 mM K_2_HPO_4_, 2 mM NaHCO_3_, pH 7.5) was added to the vial, and the suspension was homogenized. The suspension was added to a slide previously prepared with 1.5% normal melting point agarose gel. The slides were prepared with 2 gels and placed in a refrigerator for 20 min to allow the gels solidification. Then, the lysis (2.5 M NaCl, 100 mM EDTA, 10 mM Tris, 1% Triton X‐100, 10% DMSO, pH 10) was conducted for 18 h at 4°C. Following the lysis step, the slides were washed with distilled water and transferred to an electrophoresis chamber kept at 4°C. DNA unwinding was performed by incubating the slides in an alkaline solution (1 mM EDTA, 300 mM NaOH, pH > 13) for 15 min, followed by electrophoresis for 31 min at 0.64 V/cm. Subsequently, the slides were neutralized (0.4 M Tris, pH 7.5) for 15 min and fixed in ethanol for 15 min. The comets were analyzed under a fluorescence microscope after staining the slides with 0.05 nmol L^−1^ ethidium bromide. The % DNA in the tail was quantified using the Comet Score 2.0 image analysis software.

#### 2.5.3 Gene Mutation in the *Salmonella*/Microsome Test

The tests were performed using the Microplate Agar (MPA) protocol proposed by Zwarg et al. (2018), which is a miniaturized version of the *Salmonella*/microsome test in microsuspension (Kado et al. 1983). The dye was tested at concentrations ranging from 0 to 9.65 ng μL^−1^, based on its maximum solubility in DMSO. The bacterial strain used was TA1537, in which different concentrations of S9 fraction were applied (10% and 30% S9, rat liver mixture, induced by Phenobarbital/5,6‐Benzoflavone, Moltox). The negative control was DMSO, while the positive controls were 9‐aminoacridine (9AA) for assays without metabolic activation and 2‐aminoanthracene (2AA) for assays with metabolic activation.

A 50 μL aliquot of the bacterial culture and 2 μL of the samples were added to each test tube. From this mixture, 26 μL were transferred to tubes containing 25 μL of 0.015 M sodium phosphate buffer for samples without metabolic activation, or 25 μL of S9 mixture for samples with metabolic activation. The tubes were incubated at 37°C ± 1°C for 90 min under agitation and then 1 mL of top agar was added to each of them. Two hundred and fifty microliters of this content was distributed into four wells of the microplate containing 3 mL of minimal agar. Immediately after complete solidification of the medium, the microplates were incubated in a bacteriological oven at 37°C ± 1°C for 66 h. The test result was evaluated by manually counting the number of revertant colonies in the microplate wells and the viability of the bacterial strain was determined.

### 2.6 Statistical Analyses

For the chronic test with *R. subcapitata*, data normality was assessed using the Kolmogorov–Smirnov test, which indicated that the data was nonparametric. To identify significant effects and differences between treatments, the Kruskal–Wallis test with Mann–Whitney post hoc test were used, respectively. The 50% inhibitory concentration (IC_50_) and its respective 95% confidence interval (CI) were calculated when significant differences between the solvent control and the exposure concentrations were observed. This was estimated using the model fitting drm() function from the drc package (Ritz et al. 2015).

Acute toxicity data from *D. similis* , *D. rerio* , *P. hawaiensis* , and *M. galloprovincialis* were modeled by a logistic regression model to calculate the 50% effect and lethal concentration (EC_50_ and LC_50_) and their respective 95% CI, using the model fitting drm() function of the drc package (Ritz et al. 2015). Generalized linear models (GLM) assuming a normal distribution and an identity link function were used to analyze the tests conducted with *D. rerio* . All toxicity data analyses were performed in R (Ihaka and Gentleman 1996).

For both the comet and micronucleus assays, data were first tested for normality and homoscedasticity using the Shapiro–Wilk and Bartlett tests, respectively. Since comet assay data were not normally distributed, differences among treatments were analyzed using the Kruskal–Wallis ANOVA‐by‐ranks *H* followed by Dunn's post hoc test. Conversely, micronucleus data satisfied the assumptions of normality, and comparisons were performed using one‐way ANOVA (based on the *F*‐test) followed by Tukey's HSD test. These analyses were made in R (Ihaka and Gentleman 1996). Statistical significance was set at 5% for all analyses.

Analysis of variance (ANOVA), followed by linear regression using the Bernstein model (Bernstein et al. 1982), was performed for the Salmonella test using Salanal software. Results were considered positive when both ANOVA and concentration‐response analyses were significant.

## 3 Results and Discussion

### 3.1 Toxicity Tests

#### 3.1.1 Raphidocelis subcapitata

In our study, alizarin caused 17% growth inhibition in *Raphidocelis subcapitata* (72 h of exposure) at the highest concentration tested (IC_50_ > 193 μg L^−1^) (Supporting Information I—Table S3). This result is consistent with the literature, as the reported toxicity of madder extract (89.9% of alizarin) in the same test showed an IC_50_ of 8900 μg L^−1^ (Freeman et al. 2021). Our results are in agreement with a test using the plant *Lemna gibba* , which exhibited a 35% growth inhibition at 720.6 μg L^−1^ (Babu et al. 2005) and also with another study showing that alizarin affected the steady‐state electron transport in phytoplankton with an EC_50_ of 1092 ± 8.2 μg L^−1^ (Marwood et al. 2003).

#### 3.1.2 *Daphnia similis*

**FIGURE 1:**
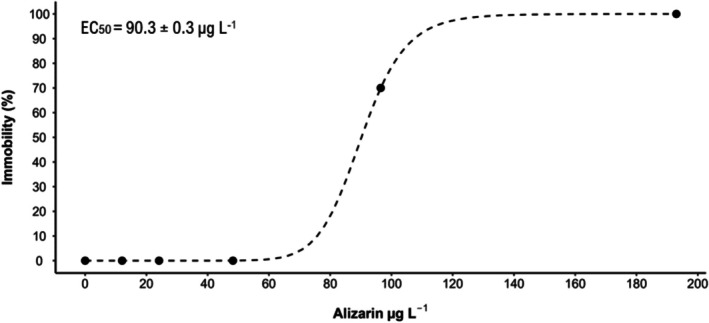
Concentration × immobility curve and effect concentration (EC_50_) of alizarin for *Daphnia similis* after 48 h of exposure.

As immobility in the control group remained below 10%, the test was considered valid. The EC_50_ of alizarin for *D. similis* was 90.3 ± 0.3 μg L^−1^ (Figure 1 and Supporting Information I—Table S4). The dye was observed inside the gut of the organism after 48 h of exposure (Supporting Information I—Figure S4). Madder extract, composed of 89.9% alizarin, showed an EC_50_ of 4400 μg L^−1^ for *D. similis* (Freeman et al. 2021).

It is known that anthraquinones absorb visible light and UV radiation, which could potentially lead to photoinduced toxicity in aquatic organisms due to the generation of reactive oxygen species during the photosensitization reaction (Wang et al. 2009). The 48 h‐EC_50_ for the constant light and dark exposure of *D. similis* to alizarin were 105 ± 55.8 μg L^−1^ and 68.6 ± 1.2 μg L^−1^, respectively (Supporting Information I—Figure S5, Tables S5 and S6). Therefore, we did not observe photoinduced toxicity, although light appeared to slightly reduce its toxicity. Our results differ from a previous study, as alizarin did not exhibit toxicity to *D. magna* under either dark or simulated solar radiation (EC_50_ > 1200 μg L^−1^) (Wang et al. 2009).

The photodegradation of alizarin was confirmed through spectrophotometric analysis. The alizarin solution prepared in *D. similis* culture medium showed absorbance peaks at 250 and 530 nm prior to exposure. Under the 16:8 h light/dark photoperiod, a slight shift in the absorbance peak was observed, exhibiting the same behavior as under dark condition (Supporting Information I—Figure S6A,B). In constant light, the peaks shifted to 270 and 570 nm, suggesting dye degradation (Supporting Information I—Figure S6C).

#### 3.1.3 *Parhyale hawaiensis*

The mortality in the control group did not exceed 10%, so the test was considered valid. Alizarin did not show acute toxicity for *P. hawaiensis* after 96 h of exposure (LC_50_ > 193 μg L^−1^) (Supporting Information I—Table S7). The dye was ingested by *P. hawaiensis*, as indicated by the presence of a red color in the digestive system (Supporting Information I—Figure S7).

#### 3.1.4 *Mytilus galloprovincialis*

The mortality in the control group did not exceed 10%, so the test was considered valid. Alizarin did not show acute toxicity for *M. galloprovincialis* after 72 h of exposure with LC_50_ > 1930 μg L^−1^ (Supporting Information I—Table S8). During the tests, it was observed that the mussels were able to filter alizarin from the exposure medium, as indicated by the gradual clearing of the water over time (Supporting Information I—Figure S8).

#### 3.1.5 *Danio rerio* (Zebrafish) Embryo Test

**FIGURE 2:**
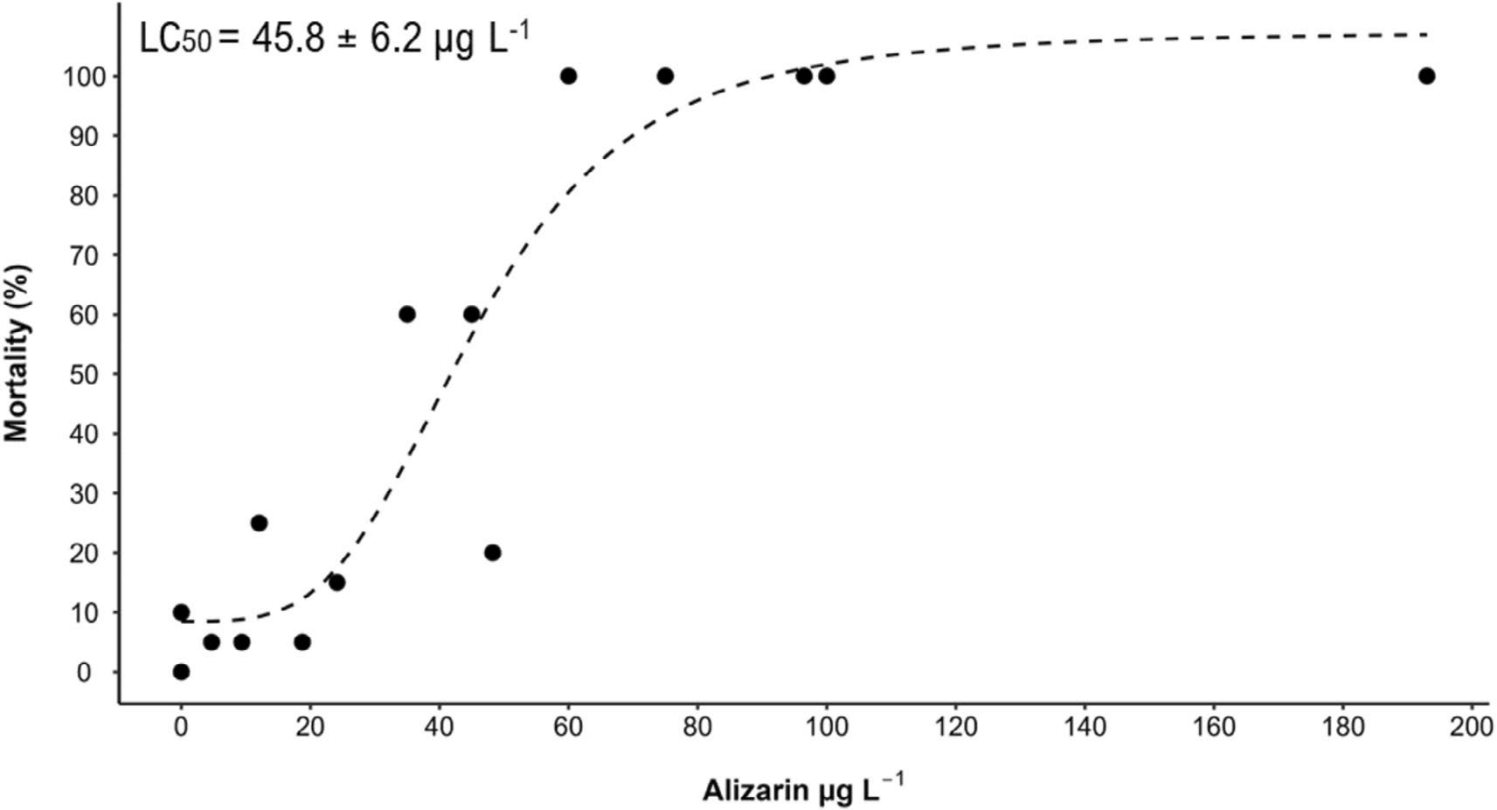
Concentration × lethality curve and lethal concentration (LC_50_) of alizarin for *Danio rerio* after 168 h of exposure.

No mortality above 10% was observed for *D. rerio* embryos in the negative and solvent controls, and embryos showed regular development. The positive control showed a mortality higher than 30%, as recommended by OECD 236 (OECD 2013). The data from the three independent tests were combined as no significant differences were found between experiments (Supporting Information I—Tables S9–S11). After 72 h, all organisms exposed to 96 μg L^−1^ of alizarin died. By 144 h, mortality reached 100% at 60 and 75 μg L^−1^ (Supporting Information I ‐ Figure S8). The 168 h LC_50_ was 45.8 ± 6.2 μg L^−1^ (Figure 2).

**FIGURE 3:**
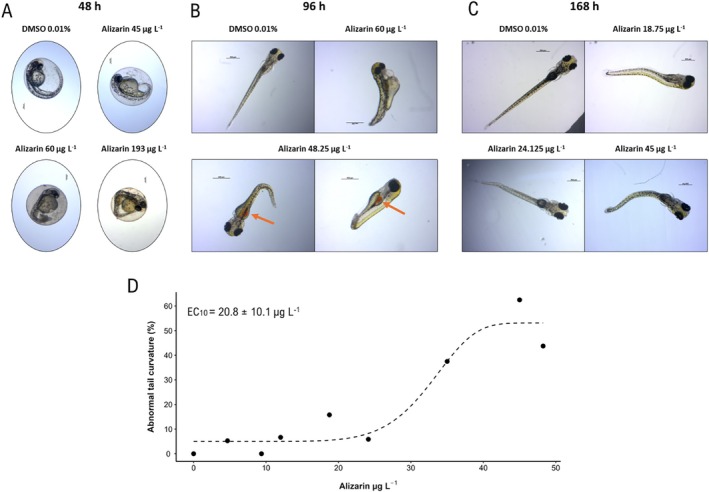
Zebrafish embryos/larvae with tail curvature at 48 h (A), 96 h (B), and 168 h (C). The orange arrow indicates alizarin inside the organism. (D) Concentration × tail curvature curve and effect concentration (EC_10_) of alizarin for *Danio rerio* embryos after 168 h of exposure. The graph and the EC_10_ were calculated based on the number of living organisms at each concentration over 168 h.

Besides mortality, we observed other sublethal effects in the experiment. Alizarin caused abnormal tail curvature after 48 h at concentrations higher than 96.5 μg L^−1^ (Figure 3A). At 96 h, all concentrations above 24.1 μg L^−1^ presented more than 10% of live embryos with abnormal tail curvature. Alizarin was observed inside the embryos at 96 h at a concentration of 48.2 μg L^−1^ (Figure 3B). The abnormal tail curvature was observed until the end of the test (Figure 3C). The EC_10_ for abnormal tail curvature was calculated as 20.8 ± 10.1 μg L^−1^ (Figure 3D).

In the literature, other studies showed that alizarin caused embryotoxicity to zebrafish. Knecht et al. (2013) observed a LC_50_ of 44,437 μg L^−1^ using dechorionated embryos 120 h post fertilization (hpf). This LC_50_ was 1000 times higher than ours. It is expected that dechorionated embryos would exhibit greater sensitivity due to the absence of the protective membrane, but this was not the case. Zebrafish embryos exposed to emodin, another anthraquinone similar to alizarin, yielded in the same range (LC_50_ of 25 μg L^−1^) (de Farias et al. 2024). Emodin (100–1500 μg L^−1^) also caused yolk sac extension, edema, and malformations of the somites and notochord (He et al. 2011).

We highlight here that this work provided reliable and relevant results of toxicity tests for alizarin, as their quality was evaluated using CRED. All tests fulfilled at least 17 of the 20 reliability criteria and 11 of the 13 relevance criteria (Supporting Information III for CRED of *R. subcapitata*, Supporting Information IV for CRED of *D. similis* , Supporting Information V for CRED of *P. hawaiensis* , Supporting Information VI for *M. galloprovincialis* and Supporting Information VII for CRED of *D. rerio* ). The two relevance criteria not satisfied are not applicable to the study. For the reliability criteria, Good Laboratory Practice (GLP) and the use of nominal concentrations were not fulfilled, except for the test with *M. galloprovincialis* , which additionally did not meet the criterion of having a standardized method. It is important to highlight that although the laboratory is not certified, it has a quality control system partially following the ISO/IEC 17025/2017.

### 3.2 Mussel Histopathology

**FIGURE 4:**
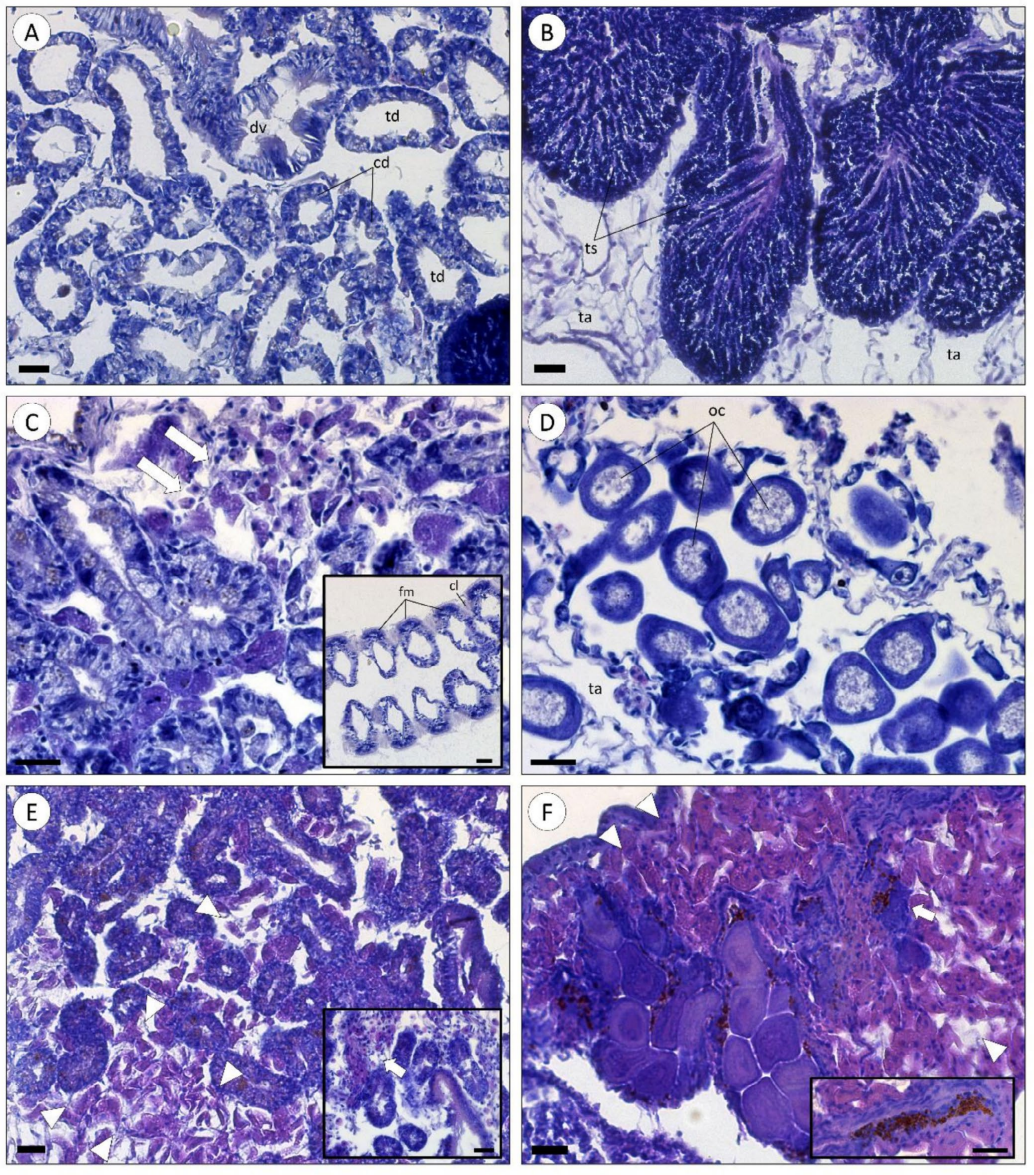
Histopathological analysis of mussels exposed to alizarin for 72 h. (A) Digestive gland of a solvent control‐exposed organism (DMSO 0.1%). The digestive tubule (diverticulum) has a normal structure, as well as digestive cells (dc). It is also possible to see the stomach diverticulum (dv), which is a tubule with ciliated and longer epithelial cells, presenting a border with microvilli. (B) Male gonad of a solvent control organism (DMSO 0.1%) with normal seminiferous tubules (ts) and adipogranular tissue (ta). (C) Digestive gland of an organism exposed to 482.5 μg L^−1^ of alizarin, showing moderate infiltration of hemocytes (white arrows). Highlight: Typical gills of an organism exposed to 482.5 μg L^−1^ of alizarin with ciliary plates (cp) binding gill filaments (fm). All concentrations yielded the expected overall structure of gills, with no evident histopathological traces. (D) Gonad of a female exposed to 965 μg L^−1^ of alizarin, with normal oocytes (oc) and adipogranular tissue (ta) showing signs of alteration. (E) Digestive gland of an organism exposed to 1930 μg L^−1^ of alizarin showing widespread infiltration of hemocytes (white triangles) and degenerated cells, revealing change in form and function. Highlight: Digestive gland of an organism exposed to 965 μg L^−1^ of alizarin, showing infiltration of hemocytes and cell degeneration (white arrow). (F) Female gonad of an organism exposed to 1930 μg L^−1^ of alizarin showing total inflammation in the adipogranular tissue (white triangles) and apoptotic oocytes (white arrow). Highlight: Increase in apoptotic cells and lipofuscin agglomerates. Scale bars: 25 μm.

The main histological alterations in mussels exposed to alizarin were found in the digestive gland and gonads (Figure 4). All alizarin concentrations caused focal hemocyte infiltration in the digestive gland, and at higher concentrations, the infiltration became widespread and cell degeneration occurred (Figure 4C,E). In the gonads, the most significant change was observed at the highest concentration tested (1930 μg L^−1^), showing diffuse inflammation accompanied by the presence of foci of apoptotic cells (Figure 4F). The gills of organisms exposed to all concentrations showed no evident histopathological features (highlighted in Figure 4C).

The digestive gland is the primary organ responsible for the metabolism of toxic compounds (Pisoni et al. 2004). Infiltration of hemocytes into the digestive gland was observed at all alizarin concentrations, particularly at the two highest concentrations (Figure 4E), where a more pronounced hemocytic infiltration was evident, indicating an active immune response. Mussel hemocytes are immune cells that can actively participate in the organism's defense against toxic compounds (Eggermont et al. 2020). Therefore, hemocyte infiltration into the digestive gland is likely a response to damage caused by alizarin exposure. Supporting this hypothesis, degeneration of the epithelial cells of the digestive gland (Figure 4C,E) was also observed, revealing histopathological alterations induced by alizarin.

At the highest alizarin concentration (1930 μg L^−1^), complete atrophy and inflammation of the gonads were observed, along with the presence of dead cells in the adipogranular tissue (highlighted in Figure 4F). Although alizarin did not cause lethality in *M. galloprovincialis* , the compound induced noticeable tissue alterations. The changes observed in the gonads are of particular concern, as they indicate that alizarin can affect a reproductive organ, potentially leading to adverse effects on the offspring.

### 3.3 Genotoxicity Effects

#### 3.3.1 Micronucleus Test With *Parhyale hawaiensis* Hemocytes

**FIGURE 5:**
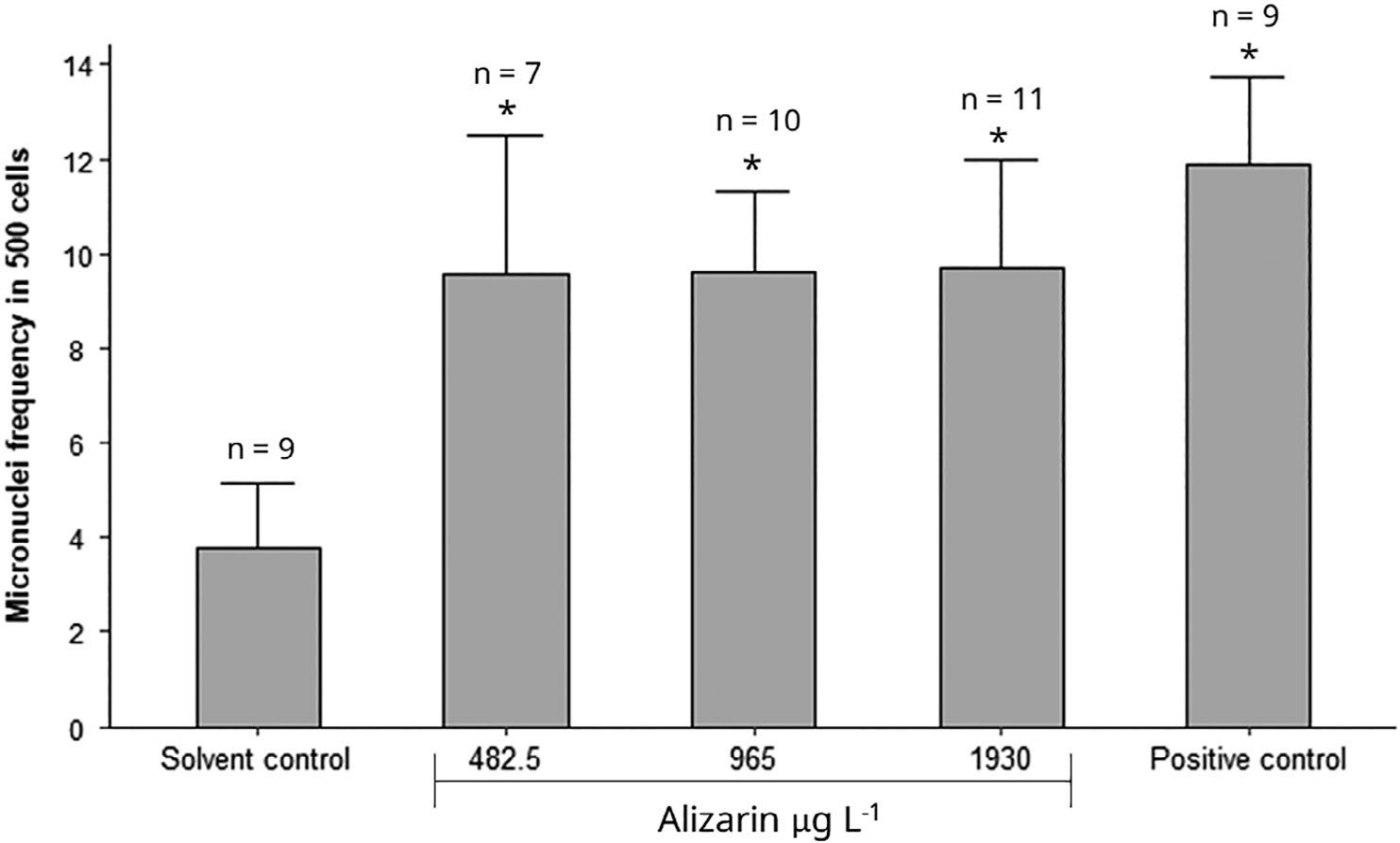
Micronucleus frequency in 500 hemocytes of *Parhyale hawaiensis* after exposure to alizarin for 96 h. Solvent control: DMSO 0.1% and positive control: Zn 1.5 mg/L. Data are presented as means + standard error of the mean; *n* represents the number of organisms analyzed in which condition and an asterisk (*) represents test conditions that were statistically different from the solvent control.

The micronucleus frequency in *P. hawaiensis* hemocytes was significantly increased after exposure to all alizarin concentrations (482.5, 965, and 1930 μg L^−1^) (Figure 5, Supporting Information—Table S12). The different alizarin concentrations tested showed a similar micronuclei frequency. Probably this high level of damage has been reached at all concentrations before 96 h exposure. As a suggestion for further studies, it would be interesting to reduce the exposure time.

Alizarin has also been shown to be mutagenic to other biological systems. Inoue et al. (2009) observed that rats fed with 0.008% and 0.04% alizarin in the basal CRF‐1 diet showed increasing preneoplastic/neoplastic lesions in the kidney, being potentially carcinogenic to this organ. A possible explanation for alizarin's carcinogenicity can be attributed to its epigenetic characteristics, due to histone modification and/or DNA methylation (Sugiyama et al. 2016). In addition, alizarin red S (ARS), a dye derived from alizarin, increased the level of intracellular reactive oxygen species (ROS) in mouse hepatocytes in vitro (Hu et al. 2019). ROS can cause DNA damage and lead to micronuclei formation (Canedo et al. 2021; Xu et al. 2014).

Other natural anthraquinones with a similar chemical structure to alizarin were also considered mutagenic. Magalhães et al. (2023) and Li et al. (2010) observed that emodin increased the micronucleus frequency in *P. hawaiensis* hemocytes and human lymphoblastoid cells, respectively. In contrast, Mengs et al. (1997) performed an in vivo test with mice and showed that emodin did not increase micronucleus frequency in bone marrow cells. Heidemann et al. (1996) showed that aloe‐emodin also did not induce micronuclei in bone marrow cells of mice exposed in vivo to this compound.

#### 3.3.2 DNA damage in *Parhyale hawaiensis* Sperm Cells

**FIGURE 6:**
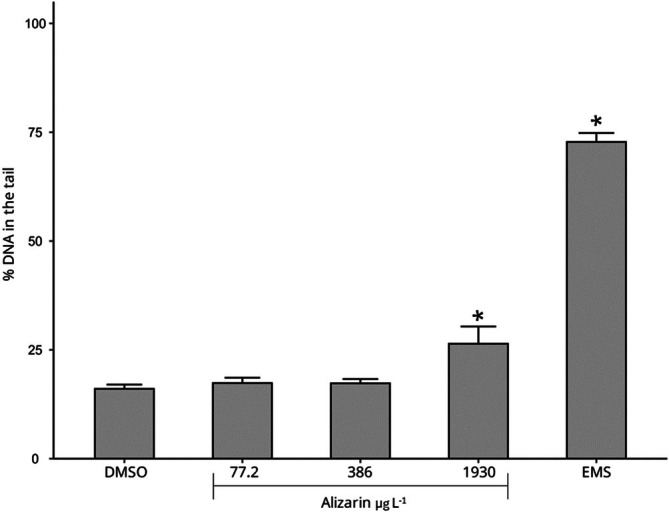
% DNA in the tail in the *Parhyale hawaiensis* sperm cells after exposure to alizarin for 96 h. DMSO is the negative control, and ethyl methanesulfonate (EMS) is the positive control. Asterisk (*) represents test conditions that were statistically different from the solvent control (TESTE, *p* < 0.05).

The sperm cells of *P. hawaiensis* exposed to 1930 μg L^−1^ showed an increase in the % DNA in the tail in comparison to the negative control (Figure 6 and Supporting Information I—Table S13). Emodin, another anthraquinone with a chemical structure similar to alizarin, has been shown to induce sperm toxicity in humans by reducing motility and decreasing the ability to penetrate a viscous medium at concentrations ranging from 27,024 to 108,096 μg L^−1^ (Luo et al. 2015). In mice, oral administration of emodin at 1000 mg kg^−1^ day^−1^ also resulted in testicular toxicity (Oshida et al. 2011). No studies were found that evaluated the toxicity of alizarin or similar anthraquinones to reproductive cells of aquatic organisms.

Alizarin has been shown to activate the aryl hydrocarbon receptor (AhR), which activates CYP enzymes that metabolize compounds, potentially increasing their toxicity (Lu et al. 2022). We believe that *P. hawaiensis* has a good metabolic capacity (Rewitz et al. 2006; Poynton et al. 2018), so we suggest that alizarin was metabolized, generating more toxic compounds that may have caused the genotoxic effect observed at the highest concentration.

In the two marine organisms used in this study, no lethal effects were observed. However, sublethal effects were detected. Both *M. galloprovincialis* and *P. hawaiensis* exhibited alterations in their reproductive organs.

#### 3.3.3 Miniaturized *Salmonella*/Microsome Test

**FIGURE 7:**
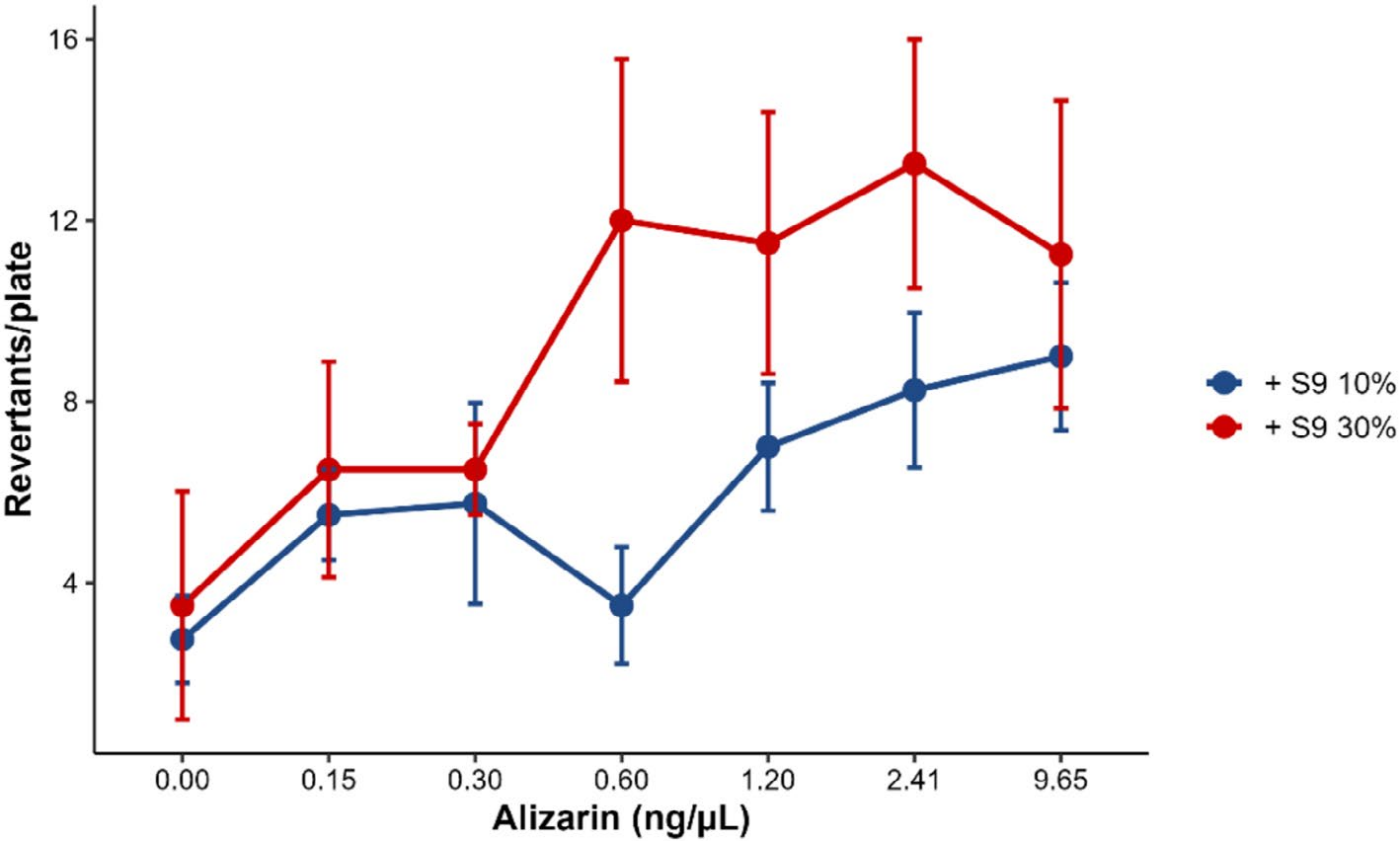
Concentration response curves for alizarin with the Salmonella strain TA1537 using 10 and 30% of S9.

Alizarin was mutagenic only in the presence of S9 (10% and 30%), with a greater response when 30% S9 was used (Figure 7 and Supporting Information—Table S14). The responsive strain TA1537 is known to be highly sensitive to anthraquinones, particularly those of natural origin (Umbuzeiro et al. 2020). The results obtained with the miniaturized *Salmonella*/microsome assay are consistent with previous studies. It has already been demonstrated that alizarin is mutagenic to TA1537 in the presence of the S9 mix (Westendorf et al. 1990; Brown and Brown 1976), as well as to other *Salmonella* strains under metabolic activation conditions (Supporting Information I—Table S2a). The MPA protocol using TA1537 with increased S9 concentrations proved to be a good alternative for testing anthraquinones, as already shown for other dyes from this group (de Farias et al. 2024; Magalhães et al. 2023; Herrala et al. 2022).

## 4 Conclusions

Alizarin was acutely toxic to *Daphnia similis* (EC_50_ = 90.3 μg L^−1^) and to *Danio rerio* embryos (LC_50_ = 45.8 μg L^−1^). The dye exhibited different sublethal effects in zebrafish embryos, with an EC_10_ of 20.8 μg L^−1^ for malformations in tail curvature. It also caused 17% growth inhibition in *Raphidocelis subcapitata* at the highest concentration tested (193 μg L^−1^). Alizarin showed no acute toxicity to *Parhyale hawaiensis* or *Mytilus galloprovincialis* . According to these results, alizarin can be classified as category 1 of the Globally Harmonized System (GHS 2023) for aquatic acute toxicity.

No data have been reported in the literature about the environmental occurrence of alizarin. But the set of data generated here allows the calculation of a reliable and relevant Predicted No‐Effect Concentration (PNEC). Once measured environmental concentratio (MEC) data are available, it will be possible to perform an appropriate risk assessment.

We confirmed that alizarin is mutagenic both in vitro (*Salmonella*/microsome assay) and in vivo, showing an increase in micronucleus frequency in *Parhyale hawaiensis* . Alizarin was more obviously toxic in the gonads of *Mytilus galloprovincialis* under the present circumstances of assessment (short‐term bioassays), causing inflammation and apoptosis. It also reached the testis of *Parhyale hawaiensis* , causing DNA damage. Prolonged chronic bioassays should be conducted in the future, especially in resilient but sensitive sentinel species like *Mytilus galloprovincialis* .

Therefore, alizarin, besides being highly toxic, is a confirmed mutagen to aquatic organisms. The results also indicate that alizarin is a potential germ cell mutagen whose effects in higher order eumetazoans, including humans, should be considered and ascertained.

## Author Contributions

Concept of the idea: Gisela de Aragão Umbuzeiro and Pedro M. Costa. Development of experiments: Amanda Rocha Rodrigues, Gabriela Cristina Fonseca Almeida, Natália Oliveira de Farias, Anjaina Fernandes de Alburquerque, Adria Caloto de Oliveira, Jessica Camila Miranda Cardoso, Gabriely Fernanda Groto Militão, Inês Moutinho Cabral, Catarina A. Faustino, João D. Vitorino, and Marina Tenório Botelho. Data analysis and interpretation of results: Amanda Rocha Rodrigues, Natália Oliveira de Farias, Anjaina Fernandes de Alburquerque, Adria Caloto de Oliveira, Marina Tenório Botelho, Pedro M. Costa, and Gisela de Aragão Umbuzeiro. Writing the manuscript: Amanda Rocha Rodrigues, Marina Tenório Botelho, and Gisela de Aragão Umbuzeiro. Review the manuscript: Gabriela Cristina Fonseca Almeida, Natália Oliveira de Farias, Anjaina Fernandes de Albuquerque, Adria Caloto de Oliveira, Inês Moutinho Cabral, Catarina A. Faustino, João D. Vitorino, and Pedro M. Costa.

## Funding

This work was supported by Fundação de Amparo à Pesquisa do Estado de São Paulo (2023/07768‐3, 2023/18005‐0, 2021/01204‐5, 2022/04482‐9), Coordenação de Aperfeiçoamento de Pessoal de Nível Superior (001), and Fundação para a Ciência e a Tecnologia (UIDP/04378/2020+UIDB/04378/2020, LA/P/0140/2020).

## Conflicts of Interest

The authors declare no conflicts of interest.

## Supporting information


**Supporting Information: I.** Control charts of *R. subcapitata*, *D. similis* , and *P. hawaiensis* , figures, data for chronic test with *R. subcapitata*, data for acute test with *D. similis* performed in 16:8 h, light and dark, data for acute test with *P. hawaiensis* , data for acute test with *Mytilus galloprovincialis* , data for genotoxicity tests (micronucleus, comet and MPA).

**Supporting Information: II.** Methodology of toxicity tests with *R. subcapitata*, *D. similis* , *P. hawaiensis* , *Mytilus galloprovincialis* , and *D. rerio* .

**Supporting Information: III.** Explanatory guidance of Criteria for Reporting and Evaluating Ecotoxicity Data (CRED).

**Supporting Information: IV.** CRED for *Raphidocelis subcapitata* chronic test.

**Supporting Information: V.** CRED for *Daphnia similis* acute test.

**Supporting Information: VI.** CRED for *Parhyale hawaiensis* acute test.

**Supporting Information: VII.** CRED for *Mytilus galloprovincialis* acute test.

**Supporting Information: VIII.** CRED for *Danio rerio* acute test.

## Data Availability

The data that supports the findings of this study are available in the Supporting Information of this article.
