# Supplementary material for: Alizarin Dye: Toxicity, Genotoxicity, and Histopathological Alterations in Model Organisms
Source: Environ Mol Mutagen. 2026 Apr 24;67(3):e70046. doi: 10.1002/em.70046 (PMC13109681; doi:10.1002/em.70046)
Supplement: Supplementary file 1 — Supporting Information: I. Control charts of R. subcapitata, D. similis , and P. hawaiensis , figures, data for chronic test with R. subcapitata, data for acute test with D. similis performed in 16:8 h, light and dark, data for acute test with P. hawaiensis , data for acute test with Mytilus galloprovincialis , data for genotoxicity tests (micronucleus, comet and MPA). [file EM-67-0-s008.docx]

**Alizarin dye: toxicity, genotoxicity and histopathological alterations in model organisms**

Amanda Rocha Rodrigues^a^, Gabriela Cristina Fonseca Almeida^a^, Natália Oliveira de Farias^a^, Anjaina Fernandes de Albuquerque^a^, Adria Caloto de Oliveira^a^, Inês Moutinho Cabral^b,c^, Catarina A. Faustino^b,c^, João D. Vitorino^b,c^, Marina Tenório Botelho^a^, Pedro M. Costa^b,c^, Gisela de Aragão Umbuzeiro^a*^

^a^ Faculdade de Tecnologia, Universidade Estadual de Campinas, Limeira, Brazil.

^b^ Associate Laboratory i4HB Institute for Health and Bioeconomy, NOVA School of Science and Technology, NOVA University of Lisbon, 2829-516 Caparica, Portugal

^c^ UCIBIO Applied Molecular Biosciences Unit, Department of Life Sciences, NOVA School of Science and Technology, NOVA University of Lisbon, 2829-516 Caparica, Portugal

^*^ Corresponding author: [giselau@unicamp.br](mailto:giselau@unicamp.br)

**
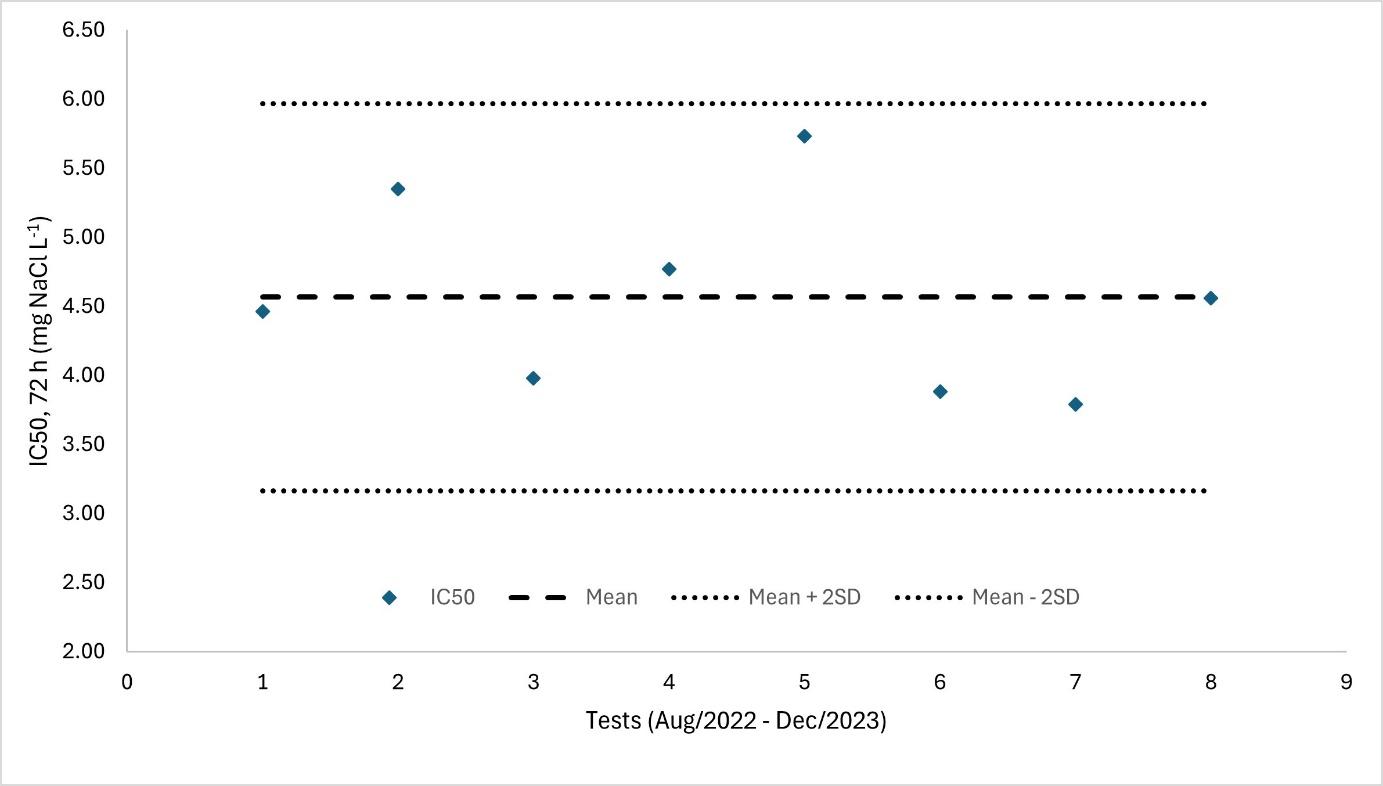
**

**Figure S1.** Control-chart of *Raphidocelis subcapitata* with acute toxicity tests using sodium chloride (NaCl) as reference toxicant during the period the chronic toxicity tests were done. SD: standard deviation; IC50: 50% inhibition concentration.

**
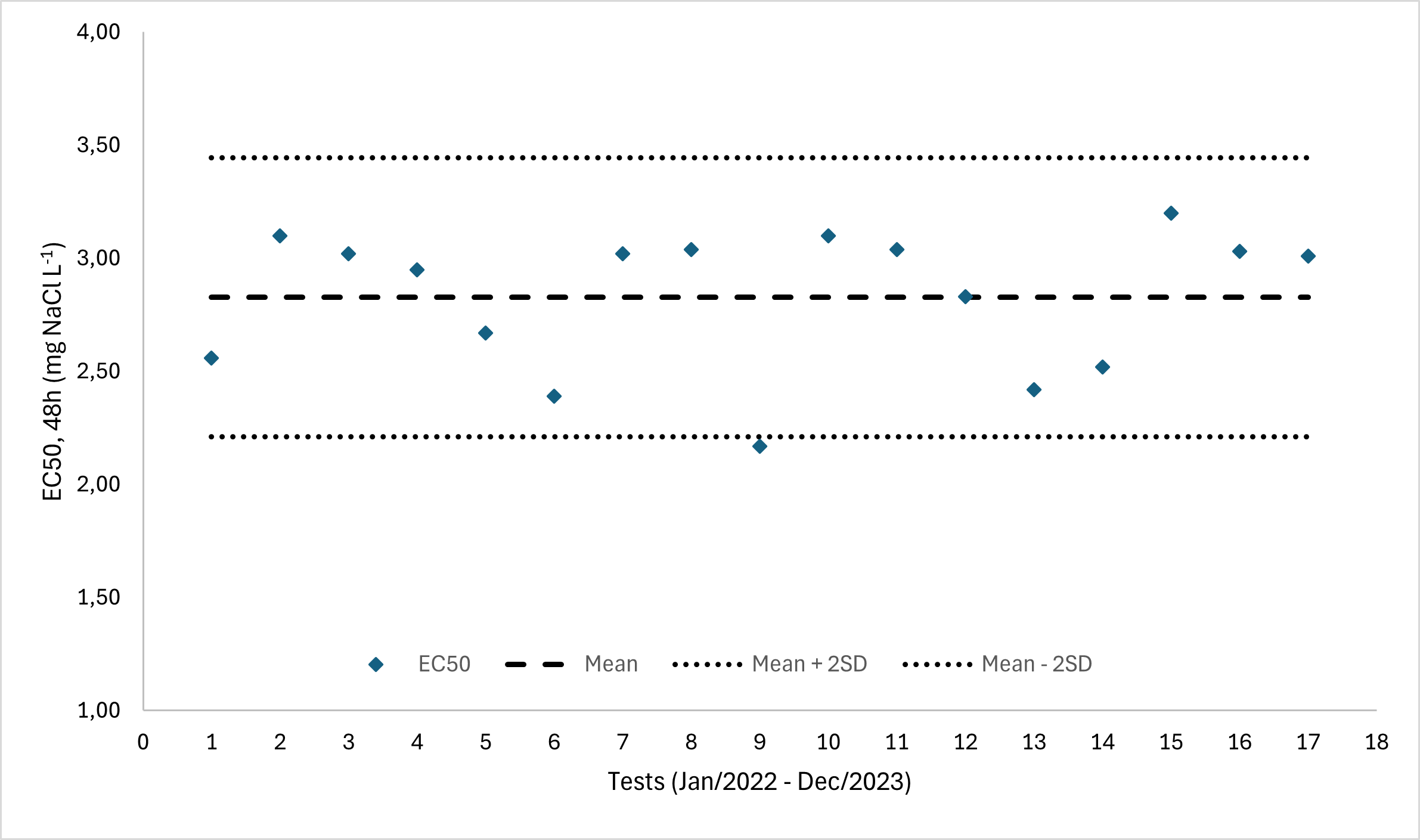
**

**Figure S2.** Control-chart of *Daphinia similis* with acute toxicity tests using sodium chloride (NaCl) as reference toxicant during the period the acute toxicity tests were done. SD: standard deviation; EC50: 50% effect concentration.

**
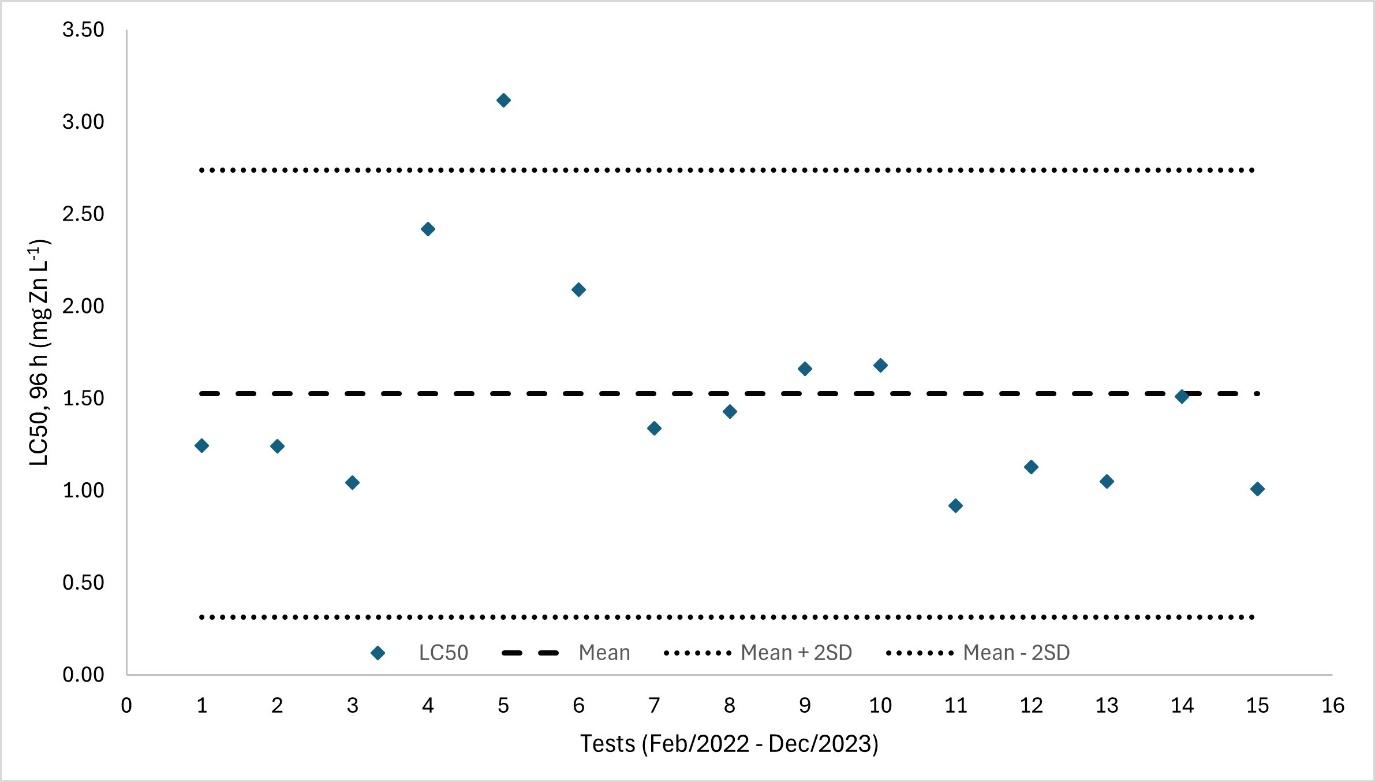
**

**Figure S3.** Control-chart of *Parhyale hawaiensis* with acute toxicity tests using Zinc (Zn) as reference toxicant during the period the acute toxicity tests were done. SD: standard deviation; LC50: 50% lethal concentration.


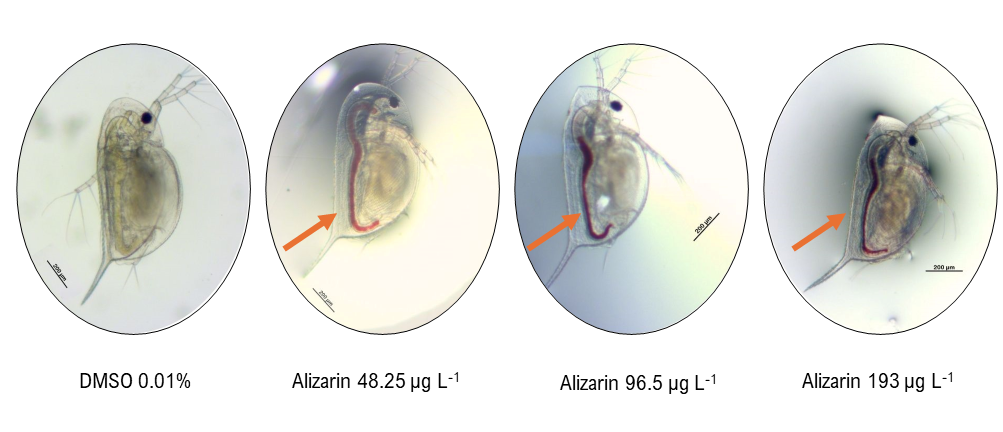


**Figure S4.** Alizarin inside the gut of *D. similis* after 48 h exposure. The orange arrow indicates the dye.


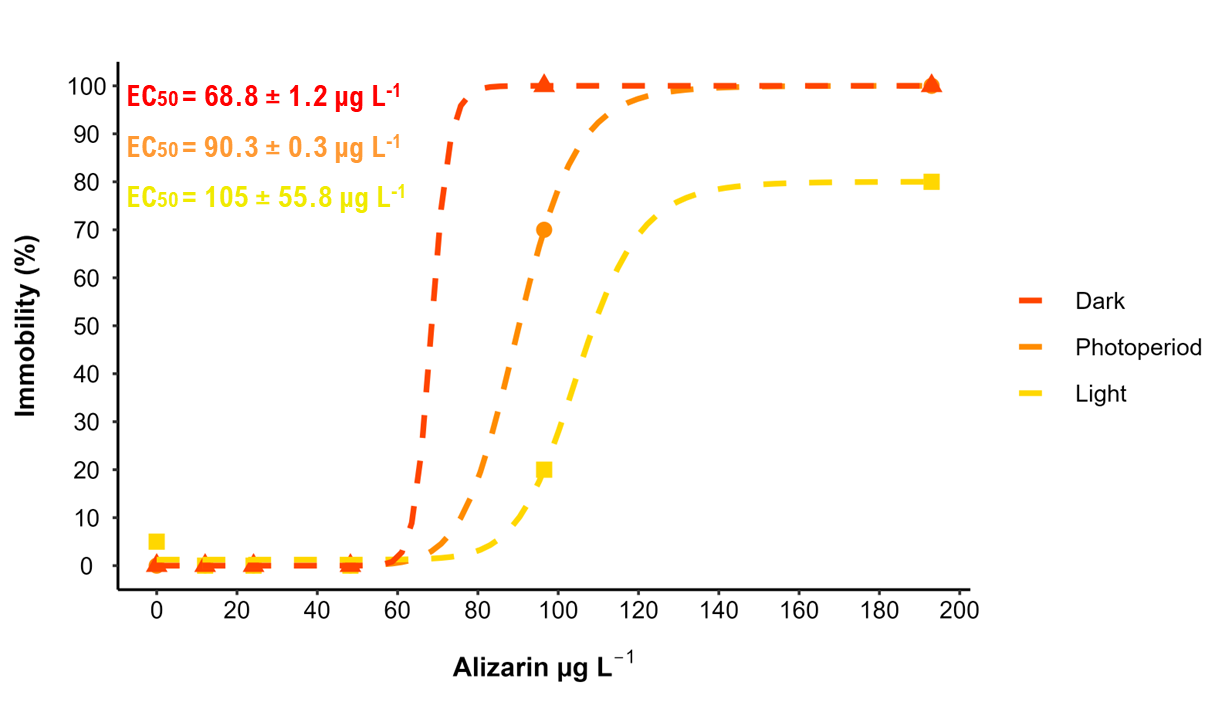


**Fig S5.** Concentration x immobility curve and effect concentration (EC_50_) of alizarin for *D. similis* exposed in dark (triangles – dark orange), photoperiod (circle - orange) and light (square – yellow).


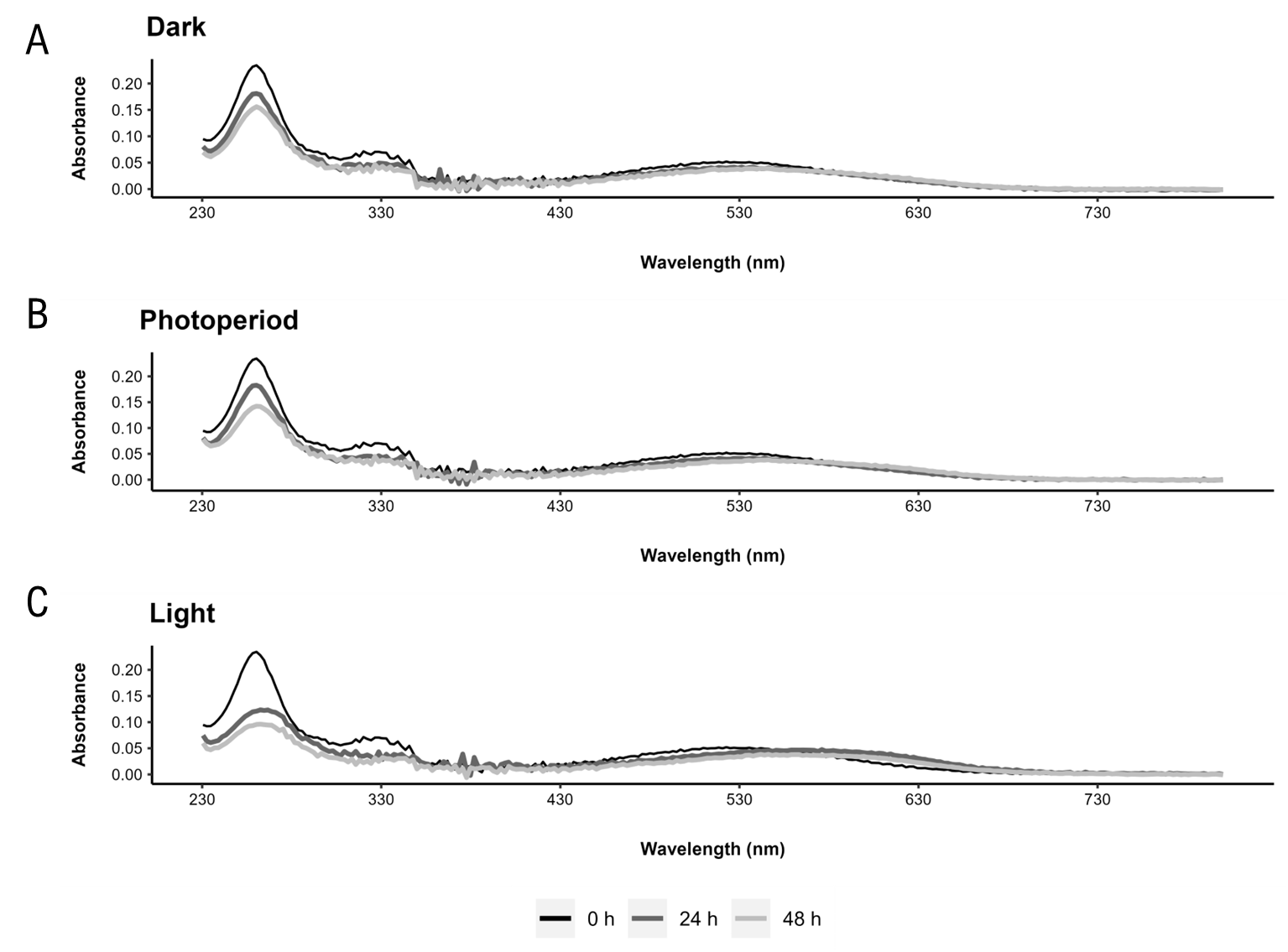


**Figure S6.** The UV-Vis spectrum of alizarin exposure for 48 h in dark (A), photoperiod (B), and light (C).


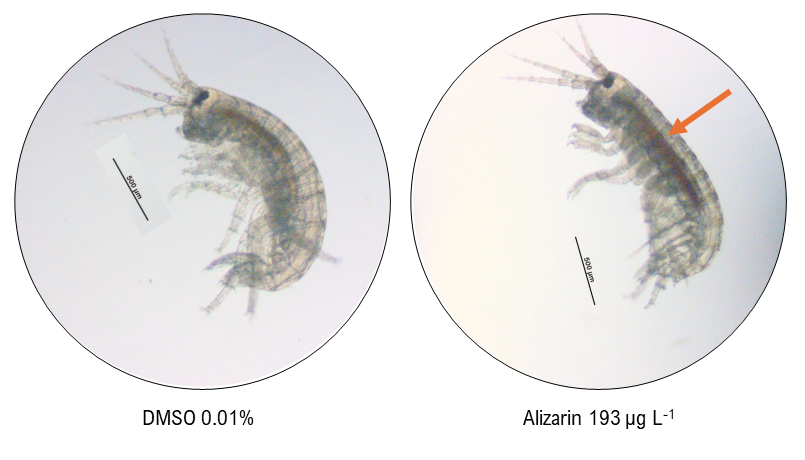


**Figure S7.** *Parhyale hawaiensis* organisms exposed to the negative control (left image) and alizarin (right image). The arrow indicates the dye inside the gut.


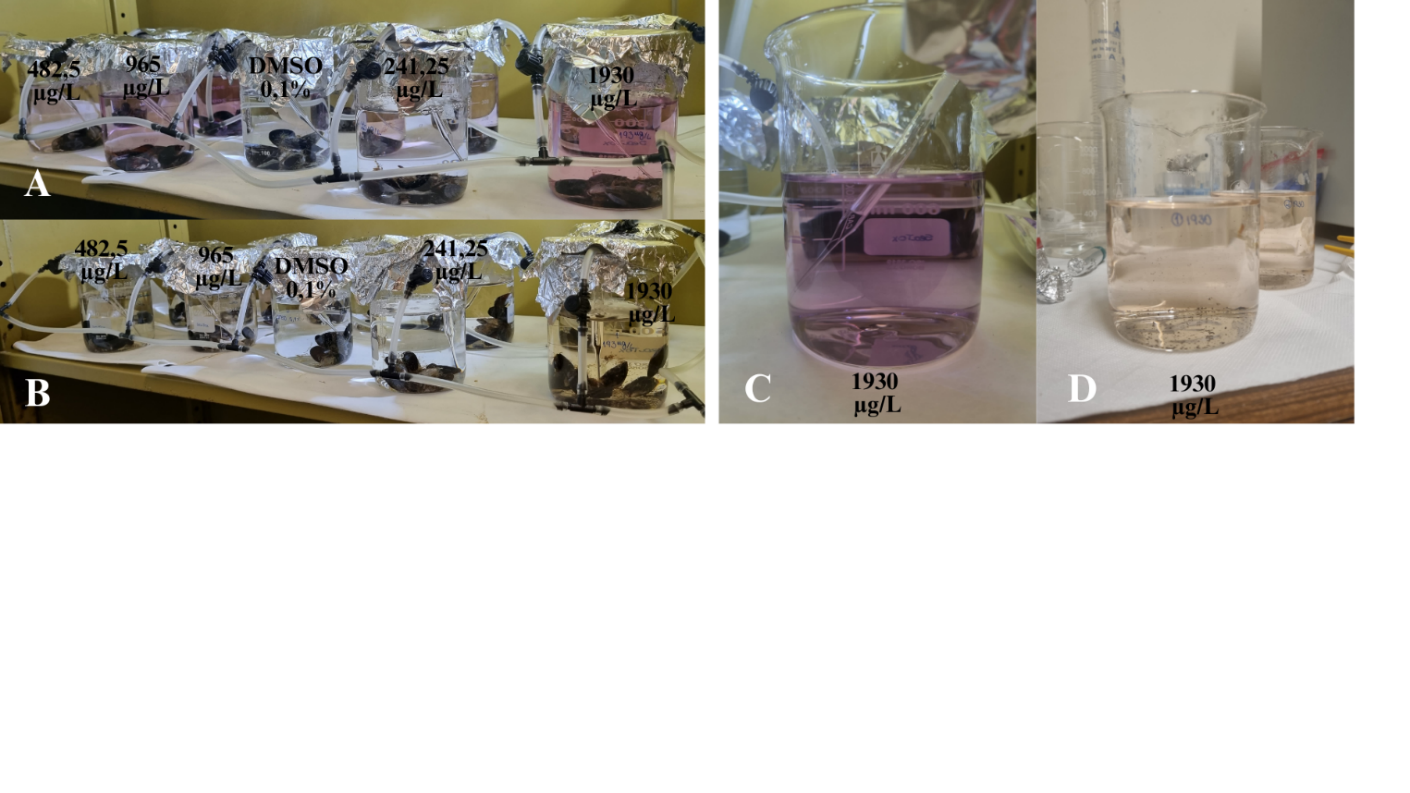
 **Figure S8.**  Acute toxicity test solutions with *Mytilus* *galloprovincialis*. A: initial moment, with alizarin dye visible in the water at higher concentrations. B: test after 24 hours of exposure, with the dye no longer visible in the water. C: highest concentration at the initial moment of the test. D: highest concentration after 24 hours of exposure, before changing the medium.


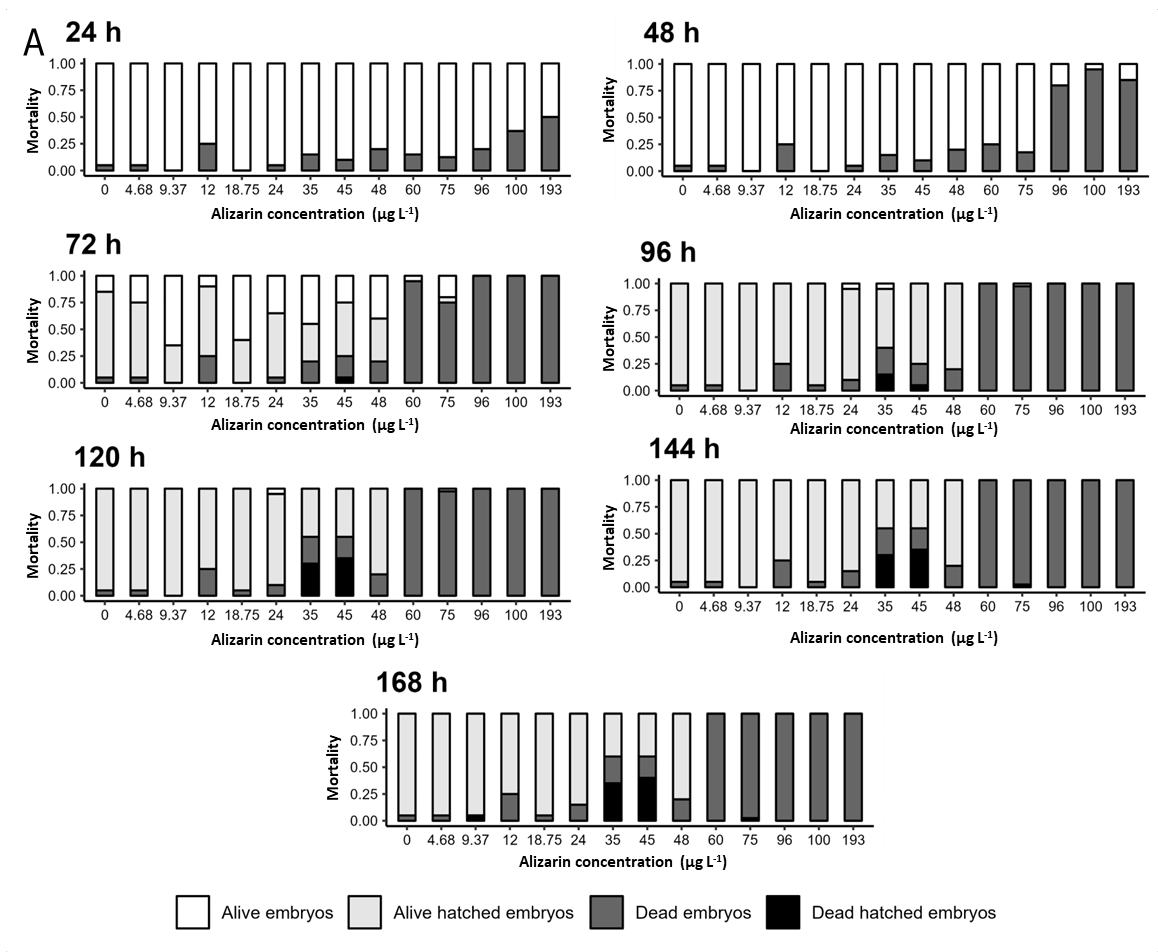


**Figure S9.**  Mortality of zebrafish embryos exposed to alizarin for 168 h. Different colors indicate dead embryos (black), alive embryos (light grey), alive hatched embryos (white) and dead hatched embryos (dark grey).

**Table S1.** Summary of the published alizarin aquatic toxicity data

| Organism | Alizarin source | Purity (%) | Endpoint/Exposure time | Results (µg L⁻¹) | Reference |
| --- | --- | --- | --- | --- | --- |
| *Lemna gibba* (plant) | Not informed | Not informed | Growth and chlorophyll levels, 8 d | Decrease of 35% in growth and 40% in chlorophyll levels in 720.6 µg L⁻¹ | Babu et al., 2005 |
| Lake Erie’s phytoplankton | Sigma Chemical | Not informed | Photosynthesis inhibition, 180 min | EC_50_ = 1,229 ± 4.9 (dark-adapted cells)  EC_50_ = 1,096 ± 8.2 (steady-state electron transport cells) | Marwood et al., 2003 |
| *Raphidocelis subcapitata* (algae) | Madder obtained from the inventory of the Wilson College of Textiles^1^ | 89.9% of alizarin | Growth inhibition, 72 h | IC_50_ = 8900 ± 400 | Freeman et al., 2021 |
| *Daphnia magma* | State Key Laboratory of Fine Chemicals^2^ | 95.7% | Immobility, 24 h | EC_50_ > 1200, dark and simulated solar radiation | Wang et al., 2009 |
| *Daphnia similis* | Madder obtained from the inventory of the Wilson College of Textiles^1^ | 89.9% of alizarin | Immobility, 48 h | EC_50_ = 4400 ± 300 Photoperiod 16h light, 8h dark | Freeman et al., 2021 |
| *Danio rerio* | Tokyo Chemical Industry | Not informed | Lethality, 120 hpf | LC_50_ = 44,437 | Knecht et al., 2013 |

^1^ North Carolina State University

^2^ Dalian University of Technology

* hpf = hour pos fertilization

**Table S2a.** Summary of the published alizarin mutagenicity data (Ames Salmonella test)

| Alizarin form and source | Purity | Concentrations | Strains | Results | Reference |
| --- | --- | --- | --- | --- | --- |
| Aglycone \| Tokyo Kasei Kogyo Co. | Not informed | 0 - 100 µg/plate | TA98 + S9 mix | - | Tikkanen et al., 1983 |
|  |  |  | TA100 + S9 mix | + |  |
|  |  |  | TA2637 + S9 mix | + |  |
| Aglycone \| Merck | Not informed | 0.1 - 100 µg/plate | TA1537 + S9 mix | + | Liberman et al., 1982 |
|  |  |  | TA1537 – S9 mix | - |  |
|  |  |  | TA102 +S9 mix | - |  |
|  |  |  | TA102 – S9 mix | - |  |
| Aglycone \| Aldrich Chemical Co. | Not informed | 0.2 – 160 µg/plate | TA98 – S9 mix | Induced mutants per nmol were determined from the midpoint of the exponential phase of the mutagenicity curve, yielding 0.037 (–S9) and 1.12 (+S9). | Westendorf et al., 1990 |
|  |  |  | TA98 + S9 mix |  |  |
|  |  |  | TA100 – S9 mix |  |  |
|  |  |  | TA100 + S9 mix |  |  |
|  |  |  | TA1535 – S9 mix |  |  |
|  |  |  | TA1537 + S9 mix |  |  |
|  |  |  | TA1537 – S9 mix |  |  |
|  |  |  | TA1537 + S9 mix |  |  |
|  |  |  | TA1538 – S9 mix |  |  |
|  |  |  | TA1538 + S9 mix |  |  |

| Table S2b. Summary of the published in vitro alizarin mutagenicity data (mammalian tests) | | | | | | | |
| --- | --- | --- | --- | --- | --- | --- | --- |
| Model organism | **Alizarin form and source** | **Purity** | **Concentrations** | **Exposure time and Endpoint** | **Results** | **Reference** |  |
| Male Wistar rats | Ruberythric acid \| C. Roth KG | Not informed | 5 – 100 µg/mL | *In vitro* exposure for 18-20 h \| Unscheduled DNA synthesis (UDS) in primary rat hepatocytes | Induced UDS in primary rat hepatocytes at 20, 50 and 100 µg/mL | Blomeke et al., 1992 |  |
| Male Wistar rats | Aglycone \| Merck | Not informed | 12.5 – 100 µg/mL | *In vitro* exposure for 18-20 h \| Unscheduled DNA synthesis (UDS) in primary rat hepatocytes | Induced UDS in primary rat hepatocytes at 50 and 100 µg/mL | Liberman et al., 1982 |  |
| Male Wistar rats | Aglycone \| Merck | Not informed | Not informed | *In vitro* exposure for 6 – 8 days \| Mutagenesis assay in V79 cells | Negative | Liberman et al., 1982 |  |
| Male Wistar rats | Aglycone \| Merck | Not informed | 3 – 100 µg/mL | *In vitro* exposure for 7 – 14 days \| Transformation of C3H/M2 Mouse Fibroblasts | No activity | Liberman et al., 1982 |  |
| Male F344 rats | Aglycone \| Merck | Not informed | 0.008% and 0.04% (m/m in the rat food) | 28 weeks \| Rat médium term multi-organ carcinogenesis bioassay | At 0.04%, it promoted atypical hyperplasias and tended to increase renal tumors | Inoue et al., 2009 |  |

**Table S3.** Data for the chronic test of Alizarin to *Raphidocelis subcapitata* performed for 72 h.

| **Concentration (**µ**g L^-1^)** | **Mean (SD)**  **(Cells mL^-1^)** | **CV (%)** | **Growth inhibition (%)** | **Statistical significance** |
| --- | --- | --- | --- | --- |
| DMSO (0.01%) | 832 (68) | 8.2 | 0.0 | - |
| 12.0625 | 1027 (22) | 2.1 | -23.5 | N. S. |
| 24.125 | 888 (33) | 3.7 | -6.7 | N. S. |
| 48.25 | 769 (90) | 11.7 | 7.5 | N. S. |
| 96.5 | 723 (36) | 5.0 | 13.1 | N. S. |
| 193 | 689 (30) | 4.4 | 17.1 | N. S. |

Mean = mean of algae cells/mL; SD = standard deviation; CV = coefficient of variation; N.S. = not statistically significant from control.

**Table S4.** Data for the acute test of Alizarin to *Daphnia similis* performed for 48 h with photoperiod.

| **Concentration**  **(ug L^-1^)** | **Number of immobilized organisms** | | | | **Total** | **Immobility (%)** |
| --- | --- | --- | --- | --- | --- | --- |
|  | **1** | **2** | **3** | **4** |  |  |
| DMSO (0.01%) | 0/5 | 0/5 | 0/5 | 0/5 | 0/20 | 0 |
| 12.0625 | 0/5 | 0/5 | 0/5 | 0/5 | 0/20 | 0 |
| 24.125 | 0/5 | 0/5 | 0/5 | 0/5 | 0/20 | 0 |
| 48.25 | 0/5 | 0/5 | 0/5 | 0/5 | 0/20 | 0 |
| 96.5 | 3/5 | 4/5 | 3/5 | 4/5 | 14/20 | 70 |
| 193.0 | 5/5 | 5/5 | 5/5 | 5/5 | 20/20 | 100 |

**Table S5.** Data for the acute test of Alizarin to *Daphnia similis* performed for 48 h with constant light.

| **Concentration**  **(ug L^-1^)** | **Number of immobilized organisms** | | | | **Total** | **Immobility (%)** |
| --- | --- | --- | --- | --- | --- | --- |
|  | **1** | **2** | **3** | **4** |  |  |
| DMSO (0.01%) | 0/5 | 1/5 | 0/5 | 0/5 | 1/20 | 5 |
| 12.0625 | 0/5 | 0/5 | 0/5 | 0/5 | 0/20 | 0 |
| 24.125 | 0/5 | 0/5 | 0/5 | 0/5 | 0/20 | 0 |
| 48.25 | 0/5 | 0/5 | 0/5 | 0/5 | 0/20 | 0 |
| 96.5 | 3/5 | 1/5 | 0/5 | 0/5 | 4/20 | 20 |
| 193.0 | 5/5 | 3/5 | 4/5 | 4/5 | 16/20 | 80 |

**Table S6.** Data for the acute test of Alizarin to *Daphnia similis* performed for 48 h with dark.

| **Concentration**  **(ug L^-1^)** | **Number of immobilized organisms** | | | | **Total** | **Immobility (%)** |
| --- | --- | --- | --- | --- | --- | --- |
|  | **1** | **2** | **3** | **4** |  |  |
| DMSO (0.01%) | 0/5 | 0/5 | 0/5 | 0/5 | 0/20 | 0 |
| 12.0625 | 0/5 | 0/5 | 0/5 | 0/5 | 0/20 | 0 |
| 24.125 | 0/5 | 0/5 | 0/5 | 0/5 | 0/20 | 0 |
| 48.25 | 0/5 | 0/5 | 0/5 | 0/5 | 0/20 | 0 |
| 96.5 | 5/5 | 5/5 | 5/5 | 5/5 | 20/20 | 100 |
| 193.0 | 5/5 | 5/5 | 5/5 | 5/5 | 20/20 | 100 |

**Table S7.** Data for the acute test of Alizarin to *Parhyale hawaiensis* performed for 96 h with photoperiod (16 h dark: 8 h light).

| **Concentration**  **(ug L^-1^)** | **Number of dead organisms** | **Total** | **Mortality (%)** |
| --- | --- | --- | --- |
| DMSO (0.01%) | 0 | 0/24 | 0 |
| 12.0625 | 0 | 0/24 | 0 |
| 24.125 | 0 | 0/24 | 0 |
| 48.25 | 0 | 0/24 | 0 |
| 96.5 | 0 | 0/24 | 0 |
| 193.0 | 0 | 0/24 | 0 |

**Table S8.** Data for the acute test of Alizarin to *Mytilus galloprovincialis*. performed for 72 h with photoperiod (14 h light: 10 h dark).

| **Concentration**  **(ug L^-1^)** | **Number of death organisms** | | | **Total** | **Mortality (%)** |
| --- | --- | --- | --- | --- | --- |
|  | **1** | **2** |  | |  |
| DMSO (0.1%) | 1/10 | 0/10 | 1/20 | | 5 |
| 241.25 | 0/10 | 0/10 | 0/20 | | 0 |
| 482.5 | 0/10 | 0/10 | 0/20 | | 0 |
| 965 | 0/10 | 0/10 | 0/20 | | 0 |
| 1930 | 0/10 | 0/10 | 0/20 | | 0 |

**Table S9.** Data for the acute test of Alizarin to *Danio rerio* embryos performed for 168 h – test 1

| **Concentration**  **(ug L^-1^)** | **Number of dead organisms** | **Total** | **Mortality (%)** |
| --- | --- | --- | --- |
| DMSO (0.01%) | 2 | 2/20 | 10 |
| 12.0625 | 5 | 5/20 | 25 |
| 24.125 | 3 | 3/20 | 15 |
| 48.25 | 4 | 4/20 | 20 |
| 96.5 | 20 | 20/20 | 100 |
| 193.0 | 20 | 20/20 | 100 |

**Table S10.** Data for the acute test of Alizarin to *Danio rerio* embryos performed for 168 h – test 2

| **Concentration**  **(ug L^-1^)** | **Number of dead organisms** | **Total** | **Mortality (%)** |
| --- | --- | --- | --- |
| DMSO (0.01%) | 2 | 2/20 | 10 |
| 35 | 5 | 12/20 | 60 |
| 45 | 12 | 12/20 | 60 |
| 60 | 20 | 20/20 | 100 |
| 75 | 20 | 20/20 | 100 |
| 100 | 20 | 20/20 | 100 |

**Table S11.** Data for the acute test of Alizarin to *Danio rerio* embryos performed for 168 h – test 3

| **Concentration**  **(ug L^-1^)** | **Number of dead organisms** | **Total** | **Mortality (%)** |
| --- | --- | --- | --- |
| DMSO (0.01%) | 0 | 0/20 | 0 |
| 4.68 | 1 | 1/20 | 5 |
| 9.37 | 1 | 1/20 | 5 |
| 18.75 | 1 | 1/20 | 5 |
| 75 | 20 | 20/20 | 100 |

**Table S12.** Data for the micronucleus test performed with *Parhyale hawaiensis* somatic cells. For each condition, 500 cells were counted.

| **Replicate** | **Condition** | **Number of micronuclei** |
| --- | --- | --- |
| 1 | 0.1% DMSO | 4 |
| 2 |  | 3 |
| 3 |  | 5 |
| 4 |  | 2 |
| 5 |  | 2 |
| 6 |  | 3 |
| 7 |  | 6 |
| 8 |  | 4 |
| 9 |  | 5 |
| 1 | Alizarin 0.48 µg/L | 15 |
| 2 |  | 5 |
| 3 |  | 10 |
| 4 |  | 9 |
| 5 |  | 9 |
| 6 |  | 9 |
| 7 |  | 10 |
| 1 | Alizarin 0.965 µg/L | 8 |
| 2 |  | 10 |
| 3 |  | 11 |
| 4 |  | 6 |
| 5 |  | 10 |
| 6 |  | 10 |
| 7 |  | 9 |
| 8 |  | 9 |
| 9 |  | 12 |
| 1 | Alizarin 1.93 µg/L | 8 |
| 2 |  | 10 |
| 3 |  | 8 |
| 4 |  | 12 |
| 5 |  | 7 |
| 6 |  | 10 |
| 7 |  | 9 |
| 8 |  | 10 |
| 9 |  | 14 |
| 10 |  | 12 |
| 11 |  | 7 |
| 1 | Zinc 1,5 mg/L | 14 |
| 2 |  | 9 |
| 3 |  | 12 |
| 4 |  | 10 |
| 5 |  | 13 |
| 6 |  | 10 |
| 7 |  | 13 |
| 8 |  | 12 |
| 9 |  | 14 |

**Table S13.** Data for the comet assay performed with *Parhyale hawaiensis* sperm cells. For each condition, 100 comets were counted.

| **Replicate** | **Gel** | **Condition** | **% DNA in tail** |
| --- | --- | --- | --- |
| 1 | 1 | 0.1% DMSO | 20.08^1^ |
|  | 2 |  | 19.86 |
| 2 | 1 |  | 15.07 |
|  | 2 |  | 13.02 |
| 3 | 1 |  | 14.22 |
|  | 2 |  | 13.12 |
| 4 | 1 |  | 16.28 |
|  | 2 |  | 16.74 |
| 1 | 1 | Alizarin 0.07 µg/L | 14.41 |
|  | 2 |  | 21.64 |
| 2 | 1 |  | 13.05 |
|  | 2 |  | 13.78 |
| 3 | 1 |  | 20.57 |
|  | 2 |  | 20.69 |
| 4 | 1 |  | 16.03 |
|  | 2 |  | 18.81 |
| 1 | 1 | Alizarin 0.38 µg/L | 15.76 |
|  | 2 |  | 14.85 |
| 2 | 1 |  | 16.36 |
|  | 2 |  | 15.00 |
| 3 | 1 |  | 20.20 |
|  | 2 |  | 17.08 |
| 4 | 1 |  | 22.88 |
|  | 2 |  | 16.33 |
| 1 | 1 | Alizarin 1.93 µg/L | 48.05^2^ |
|  | 2 |  | 25.44 |
| 2 | 1 |  | 18.15 |
|  | 2 |  | 21.31 |
| 3 | 1 |  | 19.38 |
|  | 2 |  | 21.49 |
| 4 | 1 |  | 17.94 |
|  | 2 |  | 39.45 |
| 1 | 1 | EMS | 78.01 |
|  | 2 |  | 73.04 |
| 2 | 1 |  | 77.75 |
|  | 2 |  | 71.54 |
| 3 | 1 |  | 77.80 |
|  | 2 |  | 70.09 |
| 4 | 1 |  | 73.48 |
|  | 2 |  | 60.53 |

^1^ 73 comets

^2^ 60 comets

**Table S14.** *Salmonella/*microsome assay performed with alizarin.

| **TA1537 [ ] (ng/µL)** | **With S9 10%** | | **With S9 30%** | |
| --- | --- | --- | --- | --- |
|  | **Mean** | **S.D.** | **Mean** | **S.D.** |
| 0,00 | 2,75 | 0,96 | 3,50 | 2,52 |
| 0,15 | 5,50 | 1,00 | 6,50 | 2,38 |
| 0,30 | 5,75 | 2,22 | 6,50 | 1,00 |
| 0,60 | 3,50 | 1,29 | 12,00 | 3,56 |
| 1,20 | 7,00 | 1,41 | 11,50 | 2,89 |
| 2,41 | 8,25 | 1,71 | 13,25 | 2,75 |
| 9,65 | 9,00 | 1,63 | 11,25 | 3,40 |
