# Supplementary material for: Alizarin Dye: Toxicity, Genotoxicity, and Histopathological Alterations in Model Organisms
Source: Environ Mol Mutagen. 2026 Apr 24;67(3):e70046. doi: 10.1002/em.70046 (PMC13109681; doi:10.1002/em.70046)
Supplement: Supplementary file 2 — Supporting Information: II. Methodology of toxicity tests with R. subcapitata, D. similis , P. hawaiensis , Mytilus galloprovincialis , and D. rerio . [file EM-67-0-s003.docx]

**Alizarin dye: toxicity, genotoxicity and histopathological alterations in model organisms**

Amanda Rocha Rodrigues^a^, Gabriela Cristina Fonseca Almeida^a^, Natália Oliveira de Farias^a^, Anjaina Fernandes de Albuquerque^a^, Adria Caloto de Oliveira^a^, Jessica Camila Miranda Cardoso^a^, Gabriely Fernanda Groto Militão^a^, Inês Moutinho Cabral^b,c^, Catarina A. Faustino^b,c^, João D. Vitorino^b,c^, Marina Tenório Botelho^a^, Pedro M. Costa^b,c^, Gisela de Aragão Umbuzeiro^a*^

^a^ Faculdade de Tecnologia, Universidade Estadual de Campinas, Limeira, Brazil.

^b^ Associate Laboratory i4HB Institute for Health and Bioeconomy, NOVA School of Science and Technology, NOVA University of Lisbon, 2829-516 Caparica, Portugal

^c^ UCIBIO Applied Molecular Biosciences Unit, Department of Life Sciences, NOVA School of Science and Technology, NOVA University of Lisbon, 2829-516 Caparica, Portugal

^*^ Corresponding author: [giselau@unicamp.br](mailto:giselau@unicamp.br)

**TOXICITY TEST METHODOLOGY**

1. *Raphidocelis subcapitata*

*Raphidocelis subcapitata* was maintained at 4 °C in a freshwater oligo medium. The algae inoculum was obtained from a stock sample that was incubated for 3 days before the test at 24 ± 2 °C under continuous fluorescent light (4000 ± 400 lux). This inoculum was used in a logarithmic growth phase, and to achieve exponential growth during the test, the algal biomass used was adjusted to 1 x 10^4^ cells mL^-1^. The test was performed according to the OECD 201 (2011) standardized chronic test,in Erlenmeyer flasks in triplicate. Each Erlenmeyer flask contained alizarin, the algae inoculum, and the enrichment medium, totaling a volume of 20 mL. The test was conducted for 72 h at 24 ± 2 °C under continuous light and continuous agitation (100-120 rpm) without medium renewal. Enrichment medium was used as a negative control and DMSO 0.01% as a solvent control. The concentrations of alizarin used were: 12.1, 24.1, 48.3, 96.5, and 193 μg L^-1^. Growth inhibition was measured by correlating the number of algae cells with the absorbance at 440 nm measured with a spectrophotometer (DR3900, Hach, Loveland, United States). The test was considered valid when the negative control showed cell growth at least 16 times higher.

1. *Daphnia similis*

The microcrustacean *D. similis* is cultivated in 2 L glass vessels at a temperature of 20 ± 2 °C, with a 16:8 h photoperiod (light:dark). *Daphnia similis* were fed daily with the algae *R. subcapitata,* and its culture medium (MS medium) was prepared as described in ABNT NBR:12713 (2022) and renewed three times a week.

Three acute toxicity tests were conducted on *D. similis* in accordance with OECD 202 (2004), under varying exposure conditions to evaluate the influence of light on dye toxicity. One test was performed under the photoperiod regime established by OECD 202, consisting of a 16:8 h (light:dark). In addition, exposures were conducted entirely under continuous light and continuous darkness. All tests were carried out over a 48-h period. Five neonates aged 6-24 h were used for each replicate (4 replicates per alizarin concentration), which were performed in a glass test tube. MS medium was used as a negative control, and 0.01% DMSO was used as a solvent control. Alizarin was used at 12.1, 24.1, 48.3, 96.5, and 193 μg L^-1^. The organisms were not fed and were exposed at 22 ± 2 °C. The test was considered valid when immobility in the negative and solvent control did not exceed 10%.

To complement the study of the effect of light, the absorbance spectra of the alizarin solution was measured with a UV-Vis spectrophotometer (UV-Visible Spectrometer, Cintra 6, GBC Scientific Equipment, Australia). Solutions of alizarin were prepared in the MS medium and were maintained in the same conditions as the acute toxicity tests. The absorbance was measured at three different periods of exposure: 0 h (before starting exposure), 24 h and 48 h. A sample of MS medium also exposed to the same photoperiod conditions for 48 h was used as a blank. The absorbance and wavelength measurements were used to create an absorbance graph in R.

1. *Parhyale hawaiensis*

*Parhyale hawaeinsis* was cultured according to Artal et al. (2017), in vessels containing 2 L of artificial seawater under constant aeration. The artificial seawater, prepared using deionized water and Red Sea Salt (Red Sea®), was partially renewed twice per week, with a complete water exchange performed once per month. Organisms were fed daily with commercial brands of sinking fish food. Salinity was maintained at 30 ± 2, temperature at 24 ± 2 °C and 12:12 h photoperiod (light:dark).

The acute toxicity test was performed according to Artal et al. (2017), using neonates (≤ 7 days old) individually exposed for 96 h at 24 ± 2 °C under 12:12 h photoperiod (light:dark), in 96-well microplates. Artificial seawater was used as negative control, and the solvent control was DMSO 0.01%. Alizarin concentrations were 12.1, 24.1, 48.3, 96.5, and 193 μg L^-1^. In each well, 1 neonate and 200 µL of solution were placed, and 24 replicates were prepared for each condition. After 96 h, the organisms’ mortality was assessed under a stereomicroscope (Stemi, 2000-C, Carl Zeiss, Oberkochen, Germany). The test was considered valid if the mortality of the negative and solvent controls did not exceed 10%.

1. *Mytilus galloprovincialis*

Mediterranean mussels (length: 2.87 ± 0.26 cm) were randomly collected at Costa da Caparica, W. Portugal (38°38'44.9"N 9°14'37.0"W). After collection, the organisms were transferred to the laboratory and underwent a one-week acclimatization period. The culture was mantained in aquariums with recirculating water, which was kept at 18°C with a salinity of 30 ± 2 and constant aeration, and cleaned three times a week. The mussels were fed with spirulina three times a week.

For the acute toxicity test, the organisms were exposed for 72 h to alizarin in a glass beaker at 18 °C with 14:10 h photoperiod (light:dark). The organisms were not fed during the experiment. Alizarin concentrations were 241.25, 482.5, 965, and 1930 μg L^-1^ and the solvent control was DMSO 0.1%. The exposure solutions were renewed daily. Ten mussels and 400 mL of solution were placed in each beaker and 2 replicates were prepared for each condition. Sublethal parameters in the mussels, such as valve movement and mortality, were observed every day. The test was considered valid if the mortality of the solvent control did not exceed 10%.

1. *Danio rerio* embryos

Adult fish were cultivated in aquariums with a recirculation system based on reconstituted water (reverse osmosis water plus saline salt). The water parameters were controlled: temperature was maintained at 26 ± 1 °C, photoperiod of 12:12 h (light:dark), ammonia concentration < 0.01 mg L^-1^, conductivity at 750 ± 50 µS/cm at 25 °C, pH at 7.5 ± 0.5, and dissolved oxygen above 95% saturation. The fish were fed daily with commercial food (GEMMA Micro).

The acute toxicity test was conducted in accordance with OECD 236 (2013) with 168 h of exposure for a more accurate assessment of locomotive activity. The eggs used in the experiment were collected immediately after fertilization, washed in reconstituted water (294.0 mg L^-1^ [3 mM] CaCl_2_, 123.3 mg L^-1^ [1 mM] MgSO_4_, 5.5 mg L^-1^ [0.074 mM] KCl, 63.0 mg L^-1^ [0.75 mM] NaHCO_3_ in ultrapure water), and checked under a stereomicroscope. When examined under a stereomicroscope, unfertilized eggs, and eggs with irregularities or lesions in the chorion were discarded. The eggs were exposed in 24-well microplates (one egg per well,) and 2 mL of each solution was added to each well. Four wells were used for internal control (reconstituted water). As a negative control was used reconstituted water, as a positive control was used 4 mg L^-1^ of 3,4-dichloroaniline (Sigma-Aldrich, 98% purity), and as solvent control was used DMSO 0.01% . Alizarin was tested at concentrations of 4.7 to 193 µg L^-1^ (0.02 to 0.80 μM). Three independent tests were performed, varying the exposure concentrations. The results were then combined to determine the response curve and the Lethal Concentration (LC_50_). During the test, temperature was maintained at 26 ± 1 °C, and the photoperiod was 12:12 h (light:dark). The mortality in the negative and solvent controls should not exceed 10%, while the mortality in the positive control should be more than 30% to validate the test.

The embryonic development was evaluated by daily embryos’ observation under a stereomicroscope (Stemi, 2000-C, Carl Zeiss, Oberkochen, Germany). It was evaluated lethal (coagulation, tail not detached, somite formation, no heartbeat) and sub-lethal (development of eyes, inflated swimming bladder, loss of equilibrium – larvae side-lying in the bottom of the microplate well after mechanical stimulus, eye and body pigmentation, edemas, malformation of head, tail and otoliths, deformity of yolk sac and delay in development) parameters of embryonic development.
